# Effect of salinity on the zinc(II) binding efficiency of siderophore functional groups and implications for salinity tolerance mechanisms in barley

**DOI:** 10.1038/s41598-021-95736-7

**Published:** 2021-08-18

**Authors:** George H. R. Northover, Yiru Mao, Haris Ahmed, Salvador Blasco, Ramon Vilar, Enrique Garcia-España, Dominik J. Weiss

**Affiliations:** 1 Department of Earth Science and Engineering, Imperial College London, South Kensington Campus, London, SW7 2AZ UK; 2 Instituto de Ciencia Molecular (ICMol), University of Valencia, C/Catedrático José Beltrán Martínez, 2, 46980 Paterna, Valencia Spain; 3 Department of Chemistry, Imperial College London, White City Campus, London, W12 0BZ UK; 4 Department of Civil and Environmental Engineering, Princeton University, Princeton, NJ 08540 USA

**Keywords:** Biogeochemistry, Bioinorganic chemistry, Biogeochemistry, Climate sciences, Ecology, Environmental sciences, Geochemistry

## Abstract

Bacteria, fungi and grasses use siderophores to access micronutrients. Hence, the metal binding efficiency of siderophores is directly related to ecosystem productivity. Salinization of natural solutions, linked to climate change induced sea level rise and changing precipitation patterns, is a serious ecological threat. In this study, we investigate the impact of salinization on the zinc(II) binding efficiency of the major siderophore functional groups, namely the catecholate (for bacterial siderophores), α-hydroxycarboxylate (for plant siderophores; phytosiderophores) and hydroxamate (for fungal siderophores) bidentate motifs. Our analysis suggests that the order of increasing susceptibility of siderophore classes to salinity in terms of their zinc(II) chelating ability is: hydroxamate < catecholate < α-hydroxycarboxylate. Based on this ordering, we predict that plant productivity is more sensitive to salinization than either bacterial or fungal productivity. Finally, we show that previously observed increases in phytosiderophore release by barley plants grown under salt stress in a medium without initial micronutrient deficiencies, are in line with the reduced zinc(II) binding efficiency of the α-hydroxycarboxylate ligand and hence important for the salinity tolerance of whole-plant zinc(II) status.

## Introduction

**Figure 1:**
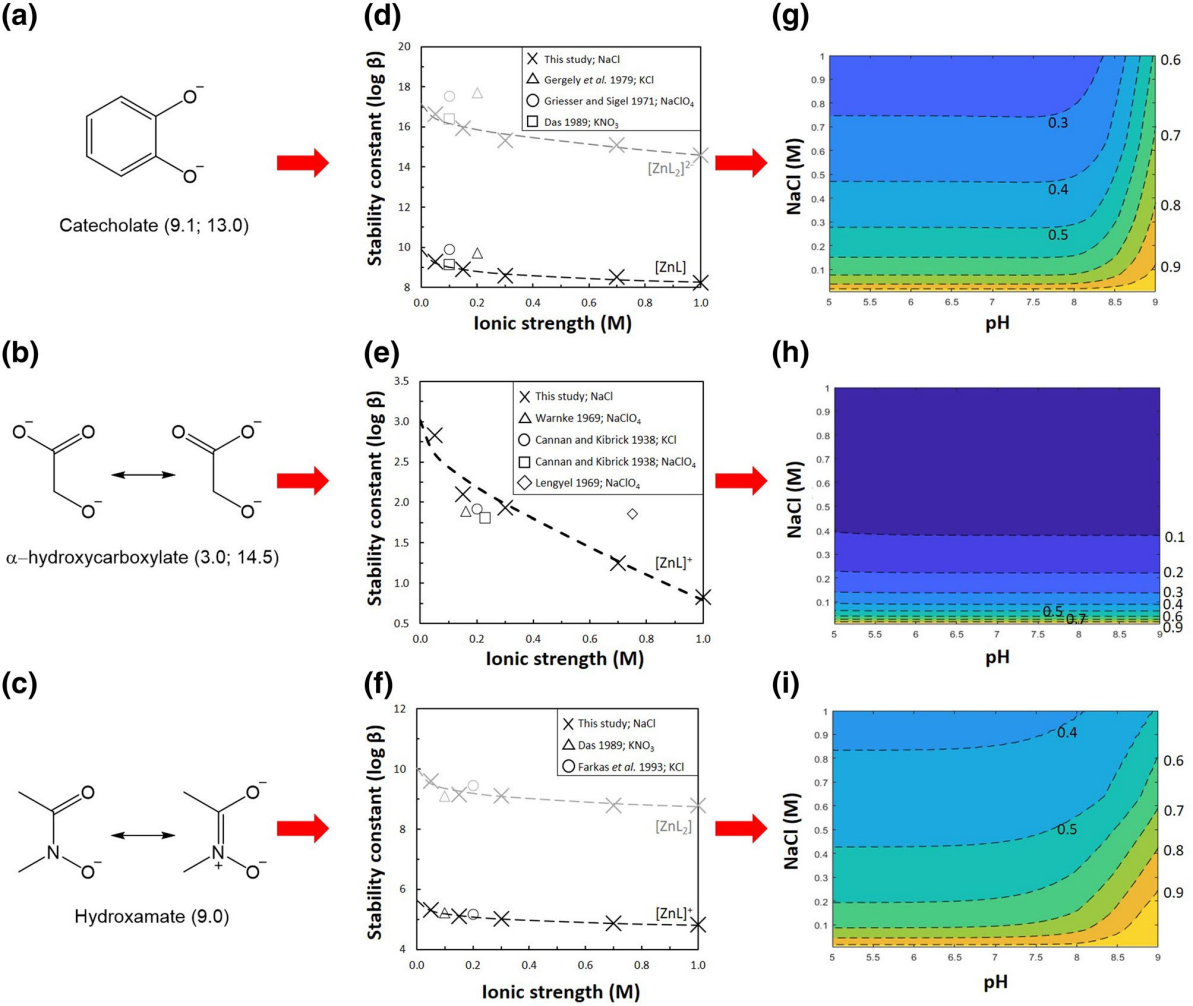
(**a–c**) Three major bidentate components of siderophores (approximate pK_a_ values). (**d-f**) Experimental zinc(II)-ligand stability constants for (**d**) pyrocatecholate, (**e**) glycolic acid, and (**f**) acetohydroxamic acid in NaCl at *T* = 298.1 K. For each species, the modified Extended Debye–Hückel model has been parameterised using experimental data from this study and is shown as a dashed line. Literature data at *T* = 298.1 K is included in the figure for comparison^50–58^. Complete set of analytical results for the zinc(II)/ligand systems, including stability constants for hydrolysed zinc(II)-ligand species, reported in Table S1. (**g-i**) Zinc(II) binding efficiency contour plots for (g) pyrocatecholate, (h) glycolic acid, and (i) acetohydroxamic acid. A zinc(II) binding efficiency value is calculated for any given pH/salinity condition by normalizing the fraction of zinc(II) complexed under these conditions, by the fraction of zinc(II) complexed by the ligand in a reference freshwater solution at the same pH. The NaCl concentration of the reference solution is equivalent to the salinity of fresh groundwater ([NaCl] = 0.005 M). Tabulated raw data is supplied in Tables S5-S7.

Organic ligands play a critical role in the cycling of trace metals. Through complexation processes, they control the mobility of contaminants and regulate the bioavailability of micronutrients^1–3^. One group of organic ligands found ubiquitously in natural solutions are siderophores^4^. Siderophores have a high affinity for iron(III) and are secreted by bacteria, fungi and grasses. Siderophores are typically hexadentate in nature, forming octahedral complexes with metal ions^5^. The three major building-blocks of siderophores are the catecholate, α-hydroxycarboxylate and hydroxamate bidentate motifs (Fig. 1a-c)^6^. Whereas the majority of bacterial siderophores contain only catecholate ligands, plant-produced siderophores (phytosiderophores) contain a mixture of functional groups, including α-aminocarboxylates and α-hydroxycarboxylate units. Hydroxamic siderophores tend to be synthesized by fungi. Model ligands featuring their functional groups are widely studied in place of real siderophores^7^. Whilst functional groups do not account for structural effects, they do capture the donor properties of siderophores, which in turn provide a significant amount of stability to metal-siderophore complexes^8^. Despite their high affinity for iron(III), siderophores are involved in the cycling of other metals^9,10^. In both bacteria and grasses, siderophores are known to function as zincophores^11–13^. For example, barley plants subjected to zinc-deficiency absorb more than 50% of their zinc(II) from soils as zinc(II)-phytosiderophore complexes^14^.

Salinization of groundwater and soil solutions is a serious, and increasingly concerning, ecological threat linked to climate change. Salinization of natural solutions is driven by sea level rise, storm surges, changes to hydrological cycles (low precipitation, high surface evaporation), weathering of native rocks and irrigation with saline water^15,16^. Freshening-up events in brackish solutions are becoming more intense in response to changing precipitation patterns^17,18^. In laboratory investigations, salinity is often represented by the concentration of NaCl ([NaCl])^19,20^. The effect of increased salinity on the complexation of zinc(II) by humic and low-molecular-weight organic acids has been studied^21–23^. For example, a sharp decrease in the proportion of zinc(II) bound to humic acid has been observed when small amounts of NaCl were added to river water (equivalent to 0.05 M NaCl)^22^. Humic acid has various functional groups with carboxylates and phenolates dominating its surface charge and reactivity^24^. Our prediction is that the ability of siderophores to bind with zinc(II) will be hindered by salinization. This is because the increased concentration of salt ions will decrease the stability of zinc(II)-siderophore complexes through: (i) activity effects and (ii) competition with chloride ions for zinc(II). If siderophores are less efficient at scavenging zinc(II) and other micronutrients, this will have fundamental implications for ecosystem productivity.

The strength of a metal–ligand complex is characterised by a stability constant. Stability constants calculated at standard state are known as intrinsic stability constants ($\log \beta^{0}$). Experimentally measured stability constants are conditional ($\log \beta$) and their value depends on the conditions under which they were measured (ionic strength, temperature and pressure)^23,25^. A conditional stability constant can be adjusted for ionic strength indirectly by calculating activity coefficients. However, indirect approaches often require ion-specific parameters to be known and have theoretical limitations which lead to a systematic bias in the adjustment of stability constants^11,26^. A more accurate, direct, method for studying the ionic strength dependence of stability constants involves measuring stability constants at a limited number of points within an ionic strength range of interest and then fitting a modified version of the Extended Debye–Hückel equation (Eq. 1) or specific ion interaction theory to the experimental data series^27–29^. We previously used a direct approach to develop an accurate description of the ionic strength dependence of stability constants for zinc(II)-citrate and zinc(II)-desferrioxamine B (DFOB) complexes^30^.

1 $$\log \beta^{0} = \log \beta - 0.51{\text{z}}^{*} \frac{{\surd {\text{I}}}}{{1 + 1.5\surd {\text{I}}}} + f\left( I \right)$$

where z is the charge of the ion, ${\text{z}}^{*} = \sum {\left( {{\text{z}}_{{{\text{reactants}}}}^{2} } \right)} - \sum {\left( {{\text{z}}_{{{\text{products}}}}^{2} } \right)}$, ${\text{I}}$ is ionic strength (M) and $f\left( I \right)$ is a linear function of ionic strength that can be formulated in different ways. The simplest expression for this term is $f\left( I \right) = CI$, where $C$ is the only adjustable parameter. Usually, this simple choice is sufficient to explain the experimental data trend in a wide ionic strength range, generally < 1.0 M.

With an accurate description of the ionic strength dependence of stability constants for zinc(II)-siderophore functional group complexes, it is possible to parameterize a two-component model (zinc(II) and ligand) for each functional group at any ionic strength, including at 0.005 M, the ionic strength representative for freshwater solutions^31,32^. A zinc(II) binding efficiency value can then be calculated for any given pH/salinity condition by normalizing the fraction of zinc(II) complexed under these conditions, by the fraction of zinc(II) complexed by the ligand in a reference freshwater solution at the same pH (Eq. 2). By comparing zinc(II) binding efficiency contour plots for siderophore functional groups over an environmentally relevant pH *vs.* salinity study region, the relative susceptibility of different siderophore classes to salinization can be established in terms of their zinc(II) chelating ability. There are few studies on the influence of ionic strength on hydrophobic interactions^33–36^. However, it has been proposed that the binding affinity of hydrophobic ligands is less affected by variations in ionic strength^37^.

2 $${\text{BE}}_{x,y} = \frac{{\left( {\frac{{{\text{ZnL}}}}{{{\text{Zn}}}}} \right)_{x,y} }}{{{ }\left( {\frac{{{\text{ZnL}}}}{{{\text{Zn}}}}} \right)_{x,z} }}$$

where ${\text{BE}}_{x,y}$ is binding efficiency at pH = $x$ and ionic strength = $y$, $\left( {\frac{{{\text{ZnL}}}}{{{\text{Zn}}}}} \right)_{x,y}$ is the fraction of zinc(II) complexed by the siderophore functional group at pH = $x$ and ionic strength = $y$ and ${ }\left( {\frac{{{\text{ZnL}}}}{{{\text{Zn}}}}} \right)_{x,z}$ is the fraction of zinc(II) complexed by the siderophore functional group at pH = $x$ and a reference ionic strength = $z$. The reference ionic strength used is equivalent to the salinity of fresh groundwater (0.005 M).

Various barley genotypes have demonstrated an ability to maintain whole-plant zinc(II) content under NaCl stress when grown in a soil or hydroponic solution without initial micronutrient deficiencies i.e., no micronutrient limitations prior to salinization (Fig. S1; Table S1)^38,39^. Moreover, hydroponic investigations where wild (*Hordeum maritimum*) and cultivated (*H. vulgare*) barley genotypes were grown in aqueous solutions without initial micronutrient deficiencies have observed that phytosiderophore release increases after a 0.10—0.20 M NaCl salinization of the growth medium^40^. One explanation for an increase in phytosiderophore release by grasses under salt stress is that it is a response to secondary micronutrient limitations induced by the disruption of uptake pathways^40^. This explanation leads to the hypothesis that previously observed increases in phytosiderophore release by barley plants grown under salt stress in a medium without initial micronutrient deficiencies, are important for the salinity tolerance of whole-plant zinc(II) status. The effectiveness of phytosiderophores at scavenging zinc(II) is linked to the stability of zinc(II)-phytosiderophore complexes. Hence, the zinc(II) binding efficiency plot for the functional group relevant for phytosiderophores can be used to estimate the increase in phytosiderophore concentration required at different salinities to maintain the overall amount of zinc(II) complexed by phytosiderophores.

The aim of this study is to quantify the effect of salinity on the zinc(II) binding efficiency of siderophore functional groups. Following this, to assess the implications for salinity tolerance mechanisms in barley.

(i) For the first time, we develop an accurate description of the ionic strength dependence of zinc(II) stability constants with catecholate (pyrocatecholate; PYR), α-hydroxycarboxylate (glycolic acid; GLY) and hydroxamate (acetohydroxamic acid; AHA) functional groups in NaCl. We do this experimentally by determining the zinc(II) stability constants with each functional group at five [NaCl] (0.05, 0.15, 0.30, 0.70, and 1.00 M) before using this data to parameterize a modified version of the Extended Debye–Hückel equation for each ligand. We test the validity of using a functional group as a representative of a real siderophore by comparing the ionic strength dependence model for zinc(II) stability constants with the hydroxamate functional group, with the ionic strength dependence model for zinc(II) stability constants with a real hydroxamate siderophore (DFOB) studied previously^30^. We highlight the benefits of the direct approach for studying the ionic strength dependence of stability constants.
(ii) Secondly, we employ the ionic strength dependence models to produce zinc(II) binding efficiency contour plots for each of the functional groups between pH 5–9 and 0.01–1.00 M NaCl. This allows us to predict which type of siderophore is most susceptible to the effects of salinity in terms of their zinc(II) chelating ability. We examine whether the susceptibility of the different classes of siderophore correlates with the hydrophobicity of the representative ligands.
(iii) Finally, we test the hypothesis that previously observed increases in phytosiderophore release by barley plants grown under salt stress in a medium without initial micronutrient deficiencies, are important for the salinity tolerance of whole-plant zinc(II) status. To achieve this objective, we use the zinc(II) binding efficiency contour plot for GLY (phytosiderophores contain carboxylate functional groups).

## Methodology and experimental set-up

### Determination of stability constants

#### Chemicals

Zinc(II) solutions were prepared by dissolving the corresponding mass of ZnCl_2_ (99%, anhydrous, VWR) in water; the exact concentration was determined by complexometric titration against ethylenediaminetetraacetic acid (EDTA) standard solutions (Fisher Scientific). Standard HCl solutions were prepared from concentrated HCl (Sigma-Aldrich-Honeywell) and standardized with tris(hydroxymethyl)aminomethane (TRIS) (Roche Diagnostics). CO_2_-free NaOH standard solutions were supplied by Fisher Scientific and were preserved from atmospheric CO_2_ by means of soda lime traps. Electrolyte solutions of NaCl were prepared from the pure salt (VWR). PYR, GLY and AHA salts (Sigma-Aldrich) were used to prepare catecholate, α-hydroxycarboxylate and hydroxamate ligand solutions, respectively. Purified water (R = 15 MΩ cm^−1^), grade A glassware and analytical grade reagents were used throughout.

#### Potentiometric titrations

Potentiometric measurements were carried out at *T* = 298.1 ± 0.1 K in thermostatted cells. The setup consisted of a Metrohm model 888 Titrando apparatus controlled by Metrohm TiAMO 1.2 software equipped with a combined gel electrode (VWR model 662 1759). Estimated precision was 0.2 mV and 0.003 ml for the electromotive force and titrant volume readings, respectively. All the potentiometric titrations were carried out under magnetic stirring and bubbling purified presaturated N_2_ through the solution to exclude O_2_ and CO_2_.

Before studying the zinc(II)/ligand systems, the protonation constants (pK_a_) of the corresponding ligand were determined at different ionic strengths (0.05 ≤ M ≤ 1.00) in NaCl solutions. A 30 ml solution containing each ligand ([L] = 5 – 10 mM), NaCl and HCl was titrated with standard NaOH solutions. For the zinc(II)/ligand systems, the titrant solutions consisted of different amounts of ligand ([L] = 5 − 10 mM), zinc(II) ([Zn] = 0.5—5 mM) and a suitable amount of HCl and NaCl. All the measurements were carried out with an excess of the ligand, with respect to zinc(II) and at different zinc(II):ligand molar ratios. The selection of ratios investigated was determined by simulating titrations using Hyperquad Simulation and Speciation computer software (HySS)^41^. [Zn] and [L] were negligible compared to the background electrolyte concentrations. Calculations showed that ionic strength remained within 10% of the targeted value throughout all titrations. To account for the influence of zinc(II) hydrolysis in the zinc(II)/ligand systems, zinc(II) titrations were carried out separately at each ionic strength to determine the hydroxide constants so that these constants could be included in the equilibrium model used to refine the titration data. In these titrations, the zinc(II) concentration in the analyte solution was 0.7 mM.

Before each experiment, independent titrations of strong acid solutions with standard base were carried out under the same medium and ionic strength conditions as the systems to be investigated, with the aim of determining the electrode potential (E^0’^) using GLEE software^42^. In this way, the pH scale used was the total scale, pH = -log [H]^+^, where [H]^+^ is the free proton concentration. For each titration, approximately 80 to 100 data points were collected. The equilibrium state during titrations was checked by confirming the time required to reach equilibrium.

#### Calculating stability constants from titration data

The software program Hyperquad was used to determine the equilibrium model and to calculate pK_a_ and stability constants from the potentiometric data set^43^. For each set of pK_a_ constants or zinc(II):ligand ratio studied, at least two repeat titrations were performed. The titration curves for each system were treated as a single set when refining the stability constants. This meant that the refinement procedure was run on both curves at the same time to derive a single set of constants. The error reported on the stability constants is the standard deviation given in the Hyperquad output file. Examples of Hyperquad files for the zinc(II)/PYR system are provided on the Zenodo repository (https://doi.org/10.5281/zenodo.4641710).

#### Ionic strength dependence of stability constants

The ionic strength dependence of the stability constants was studied with the modified version of the Extended Debye–Hückel equation^28^. The Monte Carlo method, as described by Hu et al*.*^43^, was used to estimate 95% confidence intervals on the parameters of ionic strength dependence; for each species, predicted stability constants were resampled using an inverse of the cumulative normal distribution function to give sets of simulated data from which the unknown parameters were optimized^44^. An example Excel calculation file for the application of the modified version of the Extended Debye–Hückel equation to the zinc(II)/PYR system (including the calculation of error on parameters of ionic strength dependence) has been uploaded to the Zenodo repository (https://doi.org/10.5281/zenodo.4641710).

To quantify the improvement of using a direct *vs.* indirect method for studying ionic strength dependence, the Davies equation (Eq. 3) was applied to the zinc(II)/PYR data. Speciation calculations for a [Zn] = 10^–6^ M and [L] = 10^–5^ M system at infinite dilution were conducted with the aid of HySS. The [Zn] and [L] conditions adopted here, and in the next section, are the concentrations typically used when studying the effectiveness of ligands^45,45^. The error on each speciation calculations was determined by re-running the analysis with the high/low estimates for the calculated intrinsic stability constants that can be derived from experimental data. The error was negligible and so is not shown in the associated plots.

3 $$- \log {\upgamma }_{i} = - {\text{Az}}_{i}^{2} { }\left\{ {\frac{{\surd {\text{I}}}}{{1{ } + { }\surd {\text{I}}}} - 0.3{\text{I}}} \right\}$$

where ${\text{A}}$ is a dielectric constant of the solvent, z is the charge of the ion and ${\text{I}}$ is ionic strength (M).

### Construction of zinc(II) binding efficiency contour plots

For each functional group, HySS was used to model a [Zn] = 10^–6^ M and [L] = 10^–5^ M system at 9 ionic strengths (0.005, 0.01, 0.02, 0.04, 0.08, 0.16, 0.32, 0.64, and 1.28 M NaCl). The ionic strength conditions sampled were deliberately weighted towards the lower ionic strength values, where we expect that binding efficiency is most variable. The stability constants used in the HySS models were determined by employing the ionic strength dependence models derived in the preceding phase of the investigation. The tabulated data was extracted into Excel and binding efficiencies were calculated between pH 5–9 by solving Eq. (2). This pH range was chosen because it covers the diverse conditions encountered in natural solutions^46–48^. The dataset was then imported into MATLAB and a scattered interpolation was performed to create a meshgrid which could be plotted as a 2D contoured surface map (Note S1). The use of these interpolation methods to generate a spatially continuous dataset is widespread in the environmental sciences^49^. The error on the parameters of ionic strength dependence had no effect on speciation, and thus did not influence the zinc(II) binding efficiency calculations.

### Calculation of the increase in phytosiderophore concentration required at different salinities to maintain the overall amount of zinc(II) complexed by phytosiderophores

In this section we use the zinc(II) binding efficiency contour plot for GLY. This is because phytosiderophores contain carboxylate functional groups. The zinc(II) binding efficiency of GLY was determined at 10 equally spaced intervals between 0.01 and 0.30 M NaCl at pH 5 and pH 8, respectively. The two pH values selected represented end-member conditions *i.e.*, the minimum/maximum pH that might be expected in real soil solutions^46–48^. For each pH/NaCl point, Eq. (4) was then used to calculate the theoretical increase in phytosiderophore concentration required at that pH/NaCl condition for the molar fraction of zinc(II) complexed by phytosiderophores to remain the same as in a freshwater solution at the same pH. For example, if the zinc(II) binding efficiency at the sample pH/NaCl point was 0.5, then the phytosiderophore concentration has to double to maintain the overall amount of complexed zinc(II) i.e., ${\text{R}}_{{x,y}} = 2$ .

4 $${\text{R}}_{x,y} =\left( {\frac{1}{{{\text{BE}}_{x,y} }}} \right)$$

where ${\text{BE}}_{x} ,{ }_{y}$ is the binding efficiency at pH = $x$ and ionic strength = $y$ and ${\text{R}}_{x,y}$ is the relative increase in concentration of the ligand required at pH = $x$ and ionic strength = $y$ for the molar fraction of zinc(II) complexed by the ligand to be equal to the molar fraction of zinc(II) complexed by the ligand at the same pH in a freshwater solution.

## Results and discussion

### Accurate description of the ionic strength dependence of stability constants for siderophore functional groups

Values of protonation constants for PYR, GLY and AHA (this study), and DFOB^30^, are reported at different [NaCl] and *T* = 298.1 K in Table 1 with the parameters of ionic strength dependence. Experimental and modelled zinc(II)-ligand stability constants are presented in Fig. 1d-f with literature data reported at *T* = 298.1 K in electrolyte solutions between 0.00 and 1.00 M ionic strength. Complete set of analytical results for the zinc(II)/ligand systems, including stability constants for hydrolysed zinc(II)-ligand species, reported in Table S2. Stability constants for hydrolysed zinc(II) species are reported in Table S3. The errors reported on the measured protonation/stability constants and log ${\upbeta }^{0}$ do not have an effect on subsequent speciation calculations. The errors reported on $C$ are up to an order of magnitude larger than in similar studies^29^. However, a sensitivity analysis on the three zinc(II)-ligand species with the largest relative error on $C$, shows that where log ${\upbeta }^{0}$ is recalculated for the maximum/minimum possible $C$ values, log ${\upbeta }^{0}$ remains within its error range. Hence, the error on $C$ does not affect the accuracy of log ${\upbeta }^{0}$. The error envelope for the modified Extended Debye–Hückel models in Fig. 1d-f, calculated using the uncertainty on the parameters of ionic strength dependence, was too narrow to display on the plot (less than the width of the line representing the models).

**Table 1:** Ligand protonation constants (log *β*) at different [NaCl] (M) and *T* = 298.1 K.

Ligand	Equilibrium	0.05	0.15	0.30	0.70	1.00	$\log \beta^{0}$	${\varvec{C}}$^b^
PYR	H^+^ + L^2−^ = HL^−^	12.16 ± 0.03	11.97 ± 0.01	11.94 ± .01	11.96 ± 0.01	11.79 ± 0.01	12.489 ± 0.009	0.183 ± 0.015
	2H^+^ + L^2−^ = H_2_L	21.36 ± 0.07	21.14 ± 0.02	21.06 ± 0.02	21.04 ± 0.02	20.88 ± 0.02	21.874 ± 0.001	0.296 ± 0.001
GLY	H^+^ + L^−^ = HL	3.55 ± 0.01	3.49 ± 0.01	3.54 ± 0.01	3.44 ± 0.01	3.39 ± 0.01	3.750^b^	0.070^b^
AHA	H^+^ + L^−^ = HL	9.28 ± 0.01	9.06 ± 0.01	9.10 ± 0.01	9.04 ± 0.01	9.07 ± 0.01	9.376 ± 0.001	0.085 ± 0.001
DFOB^a^	H^+^ + L^3−^ = HL^2−^	11.07 ± 0.07	10.74 ± 0.04	10.36 ± .02	10.35 ± 0.02	10.14 ± 0.06	11.491 ± 0.003	− 0.169 ± 0.003
	2H^+^ + L^3−^ = H_2_L^−^	20.94 ± 0.09	20.25 ± 0.06	19.84 ± 0.04	19.77 ± 0.04	19.77 ± 0.08	21.530 ± 0.006	0.173 ± 0.011
	3H^+^ + L^3−^ = H_3_L	30.05 ± 0.12	29.10 ± 0.09	28.61 ± 0.07	28.60 ± 0.08	28.57 ± 0.12	30.691 ± 0.012	0.194 ± 0.012
	4H^+^ + L^3−^ = H_4_L^+^	38.83 ± 0.14	37.46 ± 0.13	37.00 ± 0.10	36.97 ± 0.16	36.92 ± 0.14	39.250 ± 0.023	− 0.079 ± 0.043

^a^30.

^b^Error < 0.001 and therefore not reported.

Zinc(II)-ligand stability constants measured in this study are in good agreement with those measured in previous studies at similar ionic strengths^50–58^. For example, the 1:1 zinc(II)-GLY stability constant we report at 0.15 M NaCl differs by < 0.25 log units from the 1:1 zinc(II)-GLY stability constant reported in 0.1 M KNO_3_ and 0.2 M KCl. In agreement with previous investigations, the equilibrium model with the best statistical fit to the PYR potentiometric data comprises two zinc(II)-PYR species ([ZnL] and [ZnL_2_]^2-^) and the equilibrium model with the best statistical fit to the GLY potentiometric data comprises just one zinc(II)-GLY species ([ZnL]^+^)^50,59,60^. The stability of the GLY structure is approximately 7 $\log \beta$ units lower than the equivalent 1:1 zinc(II)-PYR structure. For the zinc(II)/AHA system, the equilibrium model with the best statistical fit to the data and makes the most chemical sense includes four zinc(II)-AHA species; [ZnL]^+^, [ZnL_2_], [Zn(OH)L] and [Zn(OH)_2_L]^−^. The stability of the 1:1 zinc(II)-AHA species lies approximately halfway between that of the equivalent PYR and GLY complexes, 3.5–4 $\log \beta$ units below/above.

The zinc(II) stability constants of all functional groups show a strong ionic strength dependence in NaCl. For PYR, GLY and AHA the 1:1 stability constant decrease by 1.67, 2.19, and 0.79 $\log {\upbeta }$ units, respectively, between 0.00 and 1.00 M NaCl. Since the absolute effect of ionic strength was of a similar order of magnitude, the relative effect is most significant in GLY which forms the weakest complexes with zinc(II). Whereas there was an almost 80% decrease in the 1:1 zinc(II)-GLY constant, the 1:1 zinc(II)-PYR constant decreases by just 20%. With respect to the validity of functional groups as representatives of real siderophores, the effect of [NaCl] on DFOB and AHA complexation is similar. The 1:1 zinc(II) stability constant for the siderophore decreases by 21% between 0.05 and 1.00 M NaCl, whereas that of the functional group decreases by 14%^30^. The ionic strength dependence parameter $C$ shows no systematic change for any of the functional groups.

**Figure 2:**
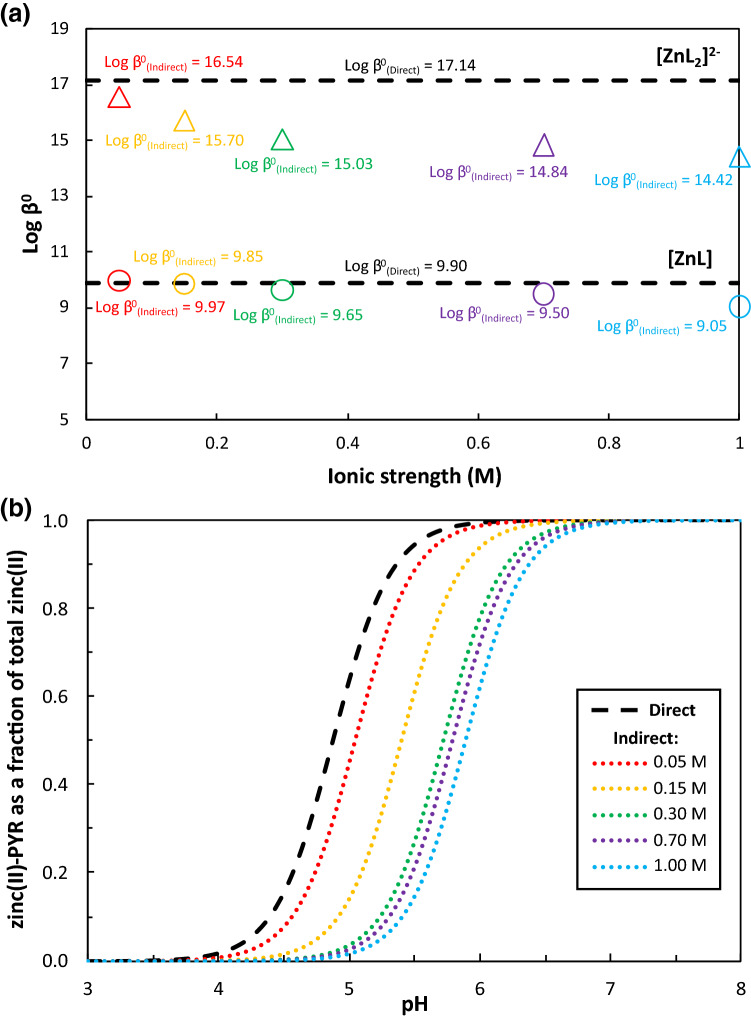
(**a**) Intrinsic stability constants for the formation of two zinc(II)-PYR (where PYR is pyrocatecholate) species ([ZnL], shown as circles, and [ZnL_2_]^2-^, shown as triangles) at different ionic strengths calculated indirectly using the Davies equation. The intrinsic stability constants for the same two species determined directly by fitting the modified version of the Extended Debye–Hückel equation to the full ionic strength dataset are shown as dashed lines. The error on the intrinsic stability constants is reported in Table S4. (**b**) Fraction of complexed zinc(II) in a [Zn] = 10^–6^ and [PYR] = 10^–5^ system modelled at infinite dilution using intrinsic stability constants determined (i) directly, by fitting the modified version of the Extended Debye–Hückel equation to the full ionic strength dataset; (ii–vi) indirectly, using the Davies equation to calculate activity coefficients and correct the zinc(II)-PYR stability constants at 0.05, 0.15, 0.30, 0.70 and 1.00 M separately.

In Fig. 2a, intrinsic stability constants for the formation of zinc(II)-PYR species ([ZnL] and [ZnL_2_]^2-^) at different ionic strengths calculated using the Davies equation are compared to the intrinsic stability constants for the same two species determined by fitting the modified version of the Extended Debye–Hückel equation to the full ionic strength dataset. Intrinsic stability constants for the full zinc(II)/PYR system calculated using the Davies equation are reported in Table S4. Figure 2b shows the fraction of complexed zinc(II) in a zinc(II)/PYR system modelled at infinite dilution using intrinsic stability constants determined (i) directly, by fitting the modified version of the Extended Debye–Hückel equation to the full ionic strength stability constant dataset (ii–vi) indirectly, using the Davies equation to calculate activity coefficients and correct the PYR stability constants separately at 0.05, 0.15, 0.30, 0.70 and 1.00 M. The disagreement between the Davies- and modified Extended Debye–Hückel-based intrinsic speciation models increases as the Davies equation is applied to higher ionic strength thermodynamic data. At pH 5, the 1.00 M Davies-based intrinsic speciation model underpredicts the fraction of complexed zinc(II) by approximately 60% compared to the modified Extended Debye–Hückel-based intrinsic speciation model. At the same pH, the 0.05 and 0.15 M Davies-based intrinsic speciation models underpredict the fraction of complexed zinc(II) by approximately 20% and 40%, respectively, compared to the modified Extended Debye–Hückel-based intrinsic speciation model. Firstly, this exercise clearly demonstrates the inconsistencies in speciation calculations that can arise when a geochemical model is parameterized using different sets of stability constants derived by applying the same indirect method (Davies equation) to different sets of ionic strength data, even when all the data is from the same study and within the activity model’s supposed ionic strength range of applicability (for Davies equation < 0.5 M i.e*.*, 0.05, 0.15 and 0.3 M). Secondly, this exercise quantifies the improvement in the accuracy of geochemical speciation calculations that can be achieved by adopting a direct method for studying the ionic strength dependence of stability constants, rather than using an indirect method, as is the common practise.

To summarise, for the first time, we have developed an accurate description of the ionic strength dependence of zinc(II) stability constants with catecholate, α-hydroxycarboxylate and hydroxamate functional groups in NaCl. All functional groups showed a strong dependence on [NaCl]. For PYR, GLY, and AHA, the 1:1 stability constant decreased by 1.67, 2.19 and 0.79 $\log \beta$ units respectively between 0.00 and 1.00 M NaCl. The overall effect of [NaCl] on DFOB, a hydroxamate siderophore, and AHA, a hydroxamate functional group, was similar. The 1:1 zinc(II) stability constant for the siderophore decreased by 21%, whereas that of the functional group decreased by 14%. We have quantified the benefits of the direct approach for studying the ionic strength dependence of stability constants; when the zinc(II)/PYR system is modelled at infinite dilution using high-precision intrinsic stability constants determined using a direct approach the accuracy of geochemical speciation calculations improves by at least 20% at pH 5 compared to when the Davies equation is used instead to calculate intrinsic stability constants.

### Comparing the susceptibility of different siderophore classes to salinity in terms of their zinc(II) chelating ability

The accurate descriptions for the ionic strength dependence of zinc(II) stability constants with siderophore functional groups developed above were subsequently employed to parameterize a two-component geochemical model (zinc(II) and ligand) for each ligand at multiple ionic strengths. Zinc(II) binding efficiency values were then calculated for specific pH/NaCl conditions by normalizing the fraction of zinc(II) complexed by the ligand at any pH/NaCl condition by the fraction of zinc(II) complexed by the ligand at the same pH in a freshwater solution (Eq. 2). Zinc(II) binding efficiency contour plots were produced for PYR, GLY and AHA between pH 5–9 and 0.01–1.00 M NaCl (Fig. 1g-i).

Changes in the concentration of NaCl have the most significant effect on the zinc(II) binding efficiency of GLY. For any pH/NaCl condition, the zinc(II) binding efficiency of GLY is lower than the zinc(II) binding efficiency of PYR or AHA. For example, at pH 7 and 0.30 M NaCl, the zinc(II) binding efficiency of GLY is between 0.1 and 0.2; this implies that at pH 7, a 0.30 M NaCl salinization of a freshwater solution causes the effectiveness of GLY for chelating zinc(II) to reduce by 80–90%. At the same pH/NaCl condition, the zinc(II) binding efficiencies of PYR and AHA are between 0.4–0.5 and 0.5–0.6, respectively. The 0.1 offset between the zinc(II) binding efficiency of PYR and AHA at pH 7 and 0.30 M NaCl is consistent across the sampling region investigated. For example, at pH 8 and 0.70 M NaCl the zinc(II) binding efficiency of PYR and AHA are between 0.3–0.4 and 0.4–0.5, respectively. This suggests that under any given pH condition, the zinc(II) chelating ability of PYR is approximately 10% more susceptible to NaCl than AHA. Hence, of the three functional groups, [NaCl] has the least significant effect on the zinc(II) binding efficiency of AHA. For GLY, in 60% of the sampling region investigated, zinc(II) binding efficiency was < 0.1. For AHA, zinc(II) binding efficiency does not drop below 0.4 under any of the pH/NaCl conditions studied.

The sensitivity of the zinc(II) binding efficiency of GLY to NaCl—demonstrated by a 60% reduction in zinc(II) binding capacity between pH 5 – 9 at 0.10 M NaCl—is similar to the effect of salinity on the zinc(II) binding capacity of humic acids previously observed^22^. The zinc(II) binding capacity of humic acid in river water decreased by 80–90% after a 0.02 M increase in NaCl; the relative zinc(II) binding capacity of humic acids remained between 10–15% across the rest of the salinity range investigated (up to approximately 0.35 M NaCl)^22^. For PYR and AHA, the contour lines are horizontal at low pH and then begin to steepen as pH increases. Where contour lines are horizontal, the effect of NaCl on zinc(II) binding efficiency is independent of pH. Where the contour lines steepen, this suggests that pH buffers the effect of NaCl on zinc(II) binding efficiency. At high pH, OH^–^ groups build-up in solution binding to zinc(II) ions, which in turn prevents the ligands under study coordinating to the metal^61,62^. The effect of OH^–^ build-up on metal–ligand interactions is independent of [NaCl]. Hence, it is not that pH is buffering the effect of NaCl, rather, that competition between the ligands under study and the OH^–^ groups is becoming increasingly important. GLY is a weak ligand and so competition with OH^–^ for zinc(II) binding sites is already maximal at a low pH. Thus, the effect of NaCl on the zinc(II) binding efficiency of GLY is independent of pH and the contour lines remain horizontal across the plot.

The hydrophobicity of a compound is represented by its partition coefficient (expressed as log P). A more negative value for log P means the compound has a higher affinity for the aqueous phase. The log P (_c_log P) values for PYR, GLY and AHA (calculated using ChemDraw v18.2) are − 5.01, − 8.76 and − 0.54, respectively. Hence, the effect of NaCl on zinc(II) binding efficiency is greatest for the most hydrophilic ligand (GLY) and smallest for the most hydrophobic ligand (AHA). This observation fits with what has previously been proposed based on the quantification of the effect of hydrophobicity on the influence of ionic strength on protein–ligand binding affinity^37^.

To summarise, analysis of the effect of [NaCl] on the zinc(II) binding efficiency of the functional groups suggests that the order of increasing susceptibility of siderophore classes to salinity in terms of their zinc(II) chelating ability is: hydroxamate < catecholate < α-hydroxycarboxylate. This trend is consistent with the relative hydrophobicity of the ligands suggesting a link between the two observations. Since α-hydroxycarboxylate ligands are associated with phytosiderophores, we predict that plant productivity is more sensitive to salinization than either bacterial or fungal productivity.

### Assessing the importance of increased phytosiderophore release as a salinity tolerance mechanism for whole-plant zinc(II) status in barley

Finally, by applying the zinc(II) binding efficiency plot for the functional group relevant for phytosiderophores (GLY), we test the hypothesis that previously observed increases in phytosiderophore release by barley plants grown under salt stress in a medium without initial micronutrient deficiencies, are important for the salinity tolerance of whole-plant zinc(II) status.

**Figure 3:**
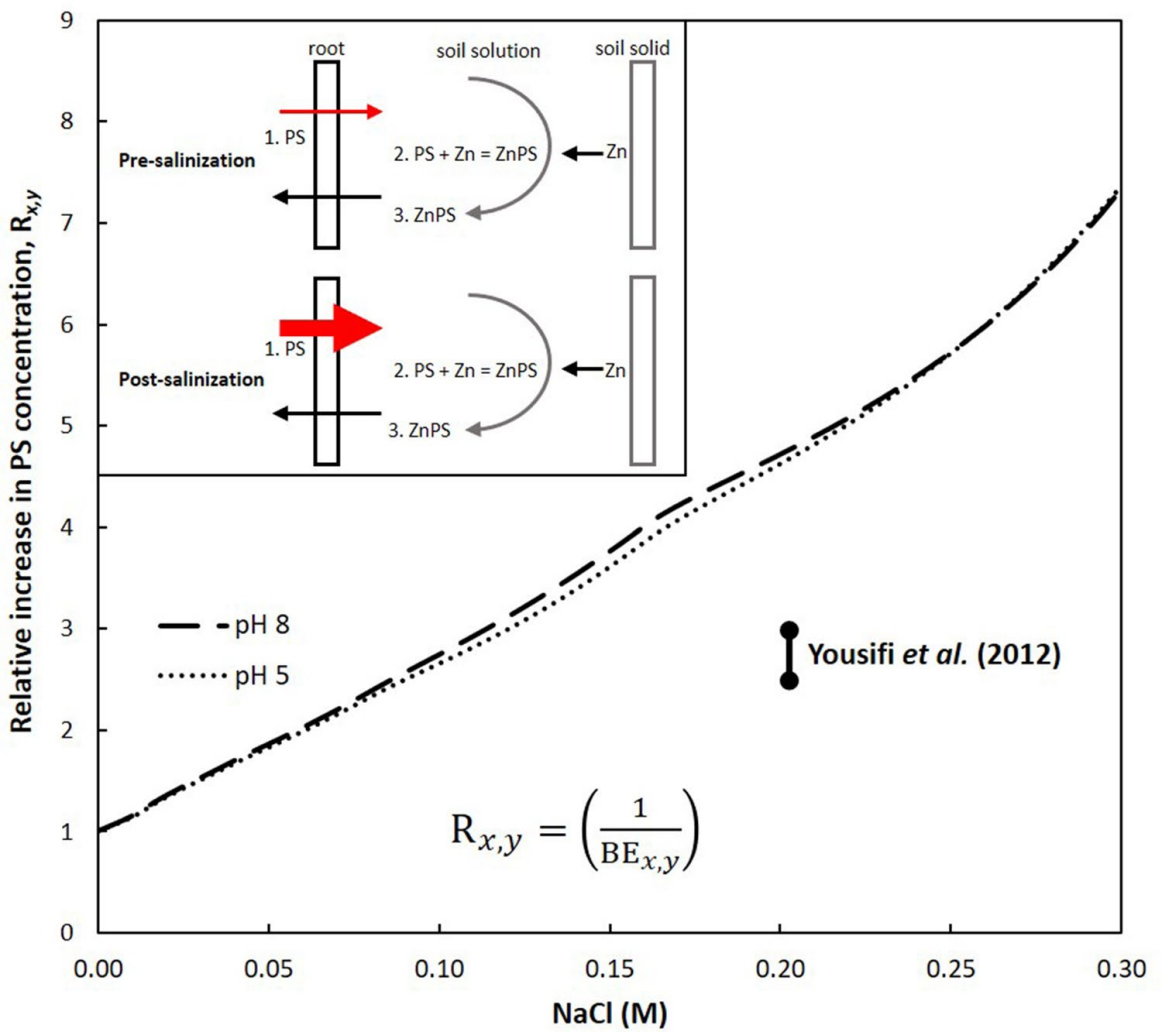
The inset gives a schematic representation of the steps involved in phytosiderophore-assisted zinc(II) acquisition and highlights the particular aspect of the pathway we focus on: (1) phytosiderophores (PS) are exuded by root cells into the surrounding environment; (2) phytosiderophores then form 1:1 water-soluble complexes with zinc(II), which may be in the soil solution, adsorbed to mineral and organic matter surfaces or present as hydroxide precipitates; and (3) the zinc(II)-phytosiderophore complexes passively diffuse back towards the root surface where the zinc(II) they have transported is assimilated into the plant. The main figure shows the calculated relative increase in phytosiderophore concentration required at different salinities for the overall amount of zinc(II) complexed by phytosiderophores to remain the same as in a freshwater solution. This is calculated using the equation displayed on the figure; where ${\text{BE}}_{x,y}$ is the binding efficiency at pH = $x$ and ionic strength = $y$ and ${\text{R}}_{x,y}$ is the relative increase in the concentration of the ligand required at pH = $x$ and ionic strength = $y$. Measurements with wild and cultivated barley plants grown in hydroponic solutions without initial micronutrient deficiencies (i.e.*,* no micronutrient limitations prior to salinization), suggest there is a 2.5–3-times increase in phytosiderophore release after 0.20 M NaCl salinization of the growth medium (this data is overlain on the plot)^40^.

The inset on Fig. 3 gives a schematic representation of the steps involved in phytosiderophore-assisted zinc(II) acquisition and highlights the particular aspect of the pathway we focus on to test our hypothesis: (step 1) phytosiderophores are exuded by root cells into the surrounding environment; (step 2) phytosiderophores form 1:1 water-soluble complexes with zinc(II), which may be in the soil solution, adsorbed to mineral and organic matter surfaces or present as hydroxide precipitates; and (step 3) the zinc(II)-phytosiderophore complexes passively diffuse back towards the root surface where the zinc(II) they have transported is assimilated into the plant^13,63–65^. Using the zinc(II) binding efficiency contour plot for GLY and Eq. (4), we can account for the effect of NaCl on the phytosiderophore-zinc(II) complexation step (step 2). This allows us to estimate how much phytosiderophore concentration, which is directly related to phytosiderophore release (step 1), must increase by at different salinities so that the overall amount of zinc(II) scavenged by the ligand and available to diffuse back towards the root-cell interface (step 3) is maintained at pre-salinization levels. We conduct our calculations at two pH conditions, representative for the pH range expected for real soil solutions^46–48.^ Fig. 3 shows that after a 0.05 M NaCl salinization of a soil solution, we would predict that a 1.8-times increase in phytosiderophore concentration is required to maintain the overall amount of zinc(II) scavenged by phytosiderophores. For phytosiderophores to scavenge the same amount of zinc(II) in a 0.30 M NaCl solution as in a freshwater solution, we estimate that phytosiderophore concentration would have to increase more than 7-times.

Previously reported experimental measurements with wild and cultivated barley plants grown in hydroponic solutions without initial micronutrient deficiencies, suggest there is a 2.5–3-times increase in phytosiderophore release at 0.20 M NaCl (data is overlain on Fig. 3)^40^. This previously observed increase in phytosiderophore release is of the same order of magnitude as the predicted increase in phytosiderophore concentration required under these conditions (calculated using the zinc(II) binding efficiency plot for GLY) to maintain the effectiveness of the uptake pathway (approximately 5-times). The calculations using the zinc(II) binding efficiency plot for GLY indicate that pH does not have a significant effect on the relative increase in phytosiderophore concentration required at different salinities; < 0.08 and > 0.24 M NaCl, there is no difference in the relative increase in phytosiderophores required at pH 5 and pH 8. The largest difference in the calculated relative increase of phytosiderophore release required to maintain the effectiveness of the uptake pathway is at 0.16 M NaCl; 3.8 and 4-times increases at pH 5 and 8, respectively. The R_*x,y*_ curves steepen as ionic strength increases, at pH 5 between 0.10 and 0.20 M NaCl ΔR_*x,y*_ = 1.9, whereas at pH 5 between 0.20 and 0.30 M NaCl ΔR_*x,y*_ = 2.8. Siderophore production is an energetically demanding process^66,67^. Salt stress is likely to make phytosiderophore production and release more challenging^68,69,70^. Given this counterpoise—the exponentially increasing demand for phytosiderophores as salinity increases *vs.* the increasing difficulty of synthesizing them—increased phytosiderophore release is most likely to be relevant for salinity tolerance at lower salinities.

To summarise, our calculation using the zinc(II) binding efficiency contour plot for GLY suggests that previously observed increases in phytosiderophore release by barley plants grown under salt stress in a medium without initial micronutrient deficiencies, are in line with the reduced zinc(II) binding efficiency of the α-hydroxycarboxylate ligand and hence important for the salinity tolerance of whole-plant zinc(II) status.

## Supplementary Information

Supplementary Information.

## Data Availability

The datasets generated during and/or analysed during the current study are available in the Zenodo repository, https://doi.org/10.5281/zenodo.4641710.
