# Supplementary material for: Effect of salinity on the zinc(II) binding efficiency of siderophore functional groups and implications for salinity tolerance mechanisms in barley
Source: Sci Rep. 2021 Aug 18;11:16704. doi: 10.1038/s41598-021-95736-7 (PMC8373983; doi:10.1038/s41598-021-95736-7)
Supplement: Supplementary file 1 — Supplementary Information. [file 41598_2021_95736_MOESM1_ESM.pdf]

Supporting Information

Effect of salinity on the zinc(II) binding efficiency of siderophore functional groups and implications for salinity tolerance mechanisms in barley

George H.R. Northover,[1*] Yiru Mao,[1] Haris Ahmed,[1] Salvador Blasco,[2] Ramon Vilar,[3] Enrique Garcia-España[2] and Dominik J. Weiss[1,4*]

[1]Department of Earth Science and Engineering, Imperial College London, South Kensington Campus, SW7 2AZ, United Kingdom

[2]Instituto de Ciencia Molecular (ICMol), University of Valencia, C/Catedrático José Beltrán Martínez, 2, 46980, Paterna, Valencia, Spain

[3]Department of Chemistry, Imperial College London, White City Campus, W12 0BZ, United Kingdom

[4]Department of Civil and Environmental Engineering, Princeton University, New Jersey 08540, United States of America

*Author(s) for correspondence: GN and DW

Contents

Table S1. Collated literature data for zinc(II) content of barley plants grown under NaCl stress in soil or hydroponic solution without initial micronutrient deficiency (*i.e.,* no micronutrient limitation prior to salinization)........................3

Table S2. Zinc(II)-ligand stability constants (log β) at different [NaCl] (M) at T = 298.1 K 6

Table S3. Zinc(II) hydrolysis constants (log β) at different [NaCl] (M) at T = 298.1 K 7

Table S4. Intrinsic stability constants (log β^0) for zinc(II)-PYR species calculated using an indirect method on data at different ionic strength (M NaCl) and a direct method on the full ionic strength dataset..8

Table S5. Concentration of zinc(II)-PYR complexes and calculated binding efficiencies at different [NaCl] (M) between pH 5 - 9..9

Table S6. Concentration of zinc(II)-GLY complexes and calculated binding efficiencies at different [NaCl] (M) between pH 5 - 9.......................................12

Table S7. Concentration of zinc(II)-AHA complexes and calculated binding efficiencies at different [NaCl] (M) between pH 5 - 9.......................................15

Figure S1. Whole-plant zinc(II) content ratio for barley plants grown under NaCl stress in soil or hydroponic solutions without initial micronutrient deficiency (*i.e.,* no micronutrient limitation prior to salinization).....................18

Note S1. Matlab code for zinc(II) binding efficiency contour plots....................19

Table S1. Collated literature data for zinc(II) content of barley plants grown under NaCl stress in soil or hydroponic solution without initial micronutrient deficiency (*i.e.,* no micronutrient limitation prior to salinization).

Reference		Cultivar	NaCl (M)	pH	Zinc(II) content ratio[a]	Error (±)[b]	Hydroponic (H) or soil (S)	Comments
1	Whole-plant	TARM-92	0.00	8.1	-		S	Seeds soaked in control solution (H_2O)
			0.09		1.07	0.02		
			0.16		1.09	0.02		
			0.24		1.17	0.02		
1	Whole-plant	TARM-92	0.00	8.1	-		S	Seeds soaked in 0.5 mM Kinetin (KIN) solution
			0.09		1.12	0.01		
			0.16		1.13	0.02		
			0.24		1.05	0.02		
1	Whole-plant	TARM-92	0.00	8.1	-		S	Seeds soaked in 2.0 mM Gibrellic acid (GA3)
			0.09		2.00	0.09		
			0.16		1.06	0.02		
			0.24		0.88	0.02		
2	Roots	CM72	0.00	6.5	-		S	Cr level = 0 µM
			0.15		0.68	0.01		
2	Roots	CM72	0.00	6.5	-		S	Cr level = 10 µM
			0.15		0.66	0.03		
2	Roots	CM72	0.00	6.5	-		S	Cr level = 50 µM
			0.15		0.82	0.05		
2	Shoots	CM72	0.00	6.5	-		S	Cr level = 0 µM
			0.15		0.79	0.05		
2	Shoots	CM72	0.00	6.5	-		S	Cr level = 10 µM
			0.15		0.82	0.12		
2	Shoots	CM72	0.00	6.5	-		S	Cr level = 50 µM
			0.15		0.98	0.11		
2	Roots	Gairdner	0.00	6.5	-		S	Cr level = 0 µM

			0.15		0.51	0.05		
2	Roots	Gairdner	0.00	6.5	-		S	Cr level = 10 µM
			0.15		0.56	0.05		
2	Roots	Gairdner	0.00	6.5	-		S	Cr level = 50 µM
			0.15		0.59	0.05		
2	Shoots	Gairdner	0.00	6.5	-		S	Cr level = 0 µM
			0.15		0.67	0.07		
2	Shoots	Gairdner	0.00	6.5	-		S	Cr level = 10 µM
			0.15		0.72	0.04		
2	Shoots	Gairdner	0.00	6.5	-		S	Cr level = 50 µM
			0.15		0.97	0.12		
3	Whole-plant	Bajwar-200	0.00	5.6	-		H	
			0.04		0.99	0.07		
			0.08		1.03	0.10		
			0.12		1.18	0.05		
4	Leaves	Iranis	0.00	7.5	-		S	350 µmol/mol CO_2 (ambient)
			0.08		0.88	0.01		
			0.16		0.94	0.02		
			0.24		1.35	0.06		
4	Leaves	Iranis	0.00	7.5	-		S	700 µmol/mol CO_2 (elevated)
			0.08		1.25	0.07		
			0.16		1.19	0.04		
			0.24		1.31	0.06		
4	Stems	Iranis	0.00	7.5	-		S	350 µmol/mol CO_2 (ambient)
			0.08		1.25	0.05		
			0.16		0.81	0.04		
			0.24		1.19	0.05		
4	Stems	Iranis	0.00	7.5	-		S	700 µmol/mol CO_2 (elevated)
			0.08		0.95	0.03		

			0.16		0.95	0.04			
			0.24		1.16	0.06			
4	Roots	Iranis	0.00	7.5	-			S	350 µmol/mol CO_2 (ambient)
			0.08		1.08	0.05			
			0.16		0.90	0.04			
			0.24		0.97	0.04			
4	Roots	Iranis	0.00	7.5	-			S	700 µmol/mol CO_2 (elevated)
			0.08		1.11	0.09			
			0.16		1.00	0.05			
			0.24		0.96	0.04			

[a]This ratio is calculated by dividing the whole-plant zinc content of the plant grown under salt stress by the whole-plant zinc(II) content of its respective control grown without NaCl added.

[b]The error reported on the literature data points was converted to a relative error. When calculating the ratio, the relative errors of the numerator and denominator were summed and then converted back into an absolute error.

Table S2. Zinc(II)-ligand stability constants (log β) at different [NaCl] (M) at T = 298.1 K.

Ligand	Equilibrium	0.05	0.15	0.30	0.70	1.00	$\log\beta^0$	C[b]
PYR	$Zn^{2+} + L^{2-} = ZnL$	9.29±0.03	8.89±0.02	8.57±0.01	8.50±0.01	8.23±0.01	9.904±0.001	0.002±0.002
	$Zn^{2+} + 2L^{2-} = ZnL_2^{2-}$	16.66±0.04	15.93±0.02	15.32±0.01	15.09±0.01	14.59±0.01	17.136±0.007	-0.924±0.012
GLY	$Zn^{2+} + L^{-} = ZnL^{+}$	2.83±0.02	2.10±0.01	1.93±0.01	1.25±0.03	0.83±0.04	3.018±0.003	-1.418±0.006
AHA	$Zn^{2+} + L^{-} = ZnL^{+}$	5.32±0.01	5.10±0.01	5.01±0.03	4.87±0.01	4.82±0.01	9.904±0.001	0.002±0.002
	$Zn^{2+} + 2L^{-} = ZnL_2$	9.60±0.01	9.16±0.01	9.11±0.01	8.79±0.01	8.79±0.01	10.021±0.001	-0.052±0.002
	$Zn^{2+} + OH^{-} + L^{-} = ZnOHL$	-3.22±0.04	-3.64±0.02	-4.22±0.08	-4.07±0.02	-4.41±0.04	-3.683±0.017	1.2095±0.027
	$Zn^{2+} + 2OH^{-} + L^{-} = ZnOH_2L^{-}$	-13.60±0.04	-13.65±0.01	-13.21±0.07	-14.07±0.02	-13.96±0.01	-13.446±0.011	0.571±0.017
DFOB[a]	$Zn^{2+} + L^{3-} = ZnL^{-}$	11.67±0.06	9.91±0.02	9.97±0.04	9.39±0.05	9.32±0.04	12.027±0.039	-0.486±0.077
	$Zn^{2+} + H^{+} + L^{3-} = ZnHL$	21.55±0.11	19.85±0.04	19.85±0.06	19.34±0.09	19.38±0.06	22.105±0.026	-0.075±0.053
	$Zn^{2+} + 2H^{+} + L^{3-} = ZnH_2L^{+}$	29.30±0.13	27.81±0.05	27.42±0.08	27.19±0.12	27.25±0.08	29.872±0.027	0.009±0.051

[a]5

Table S3. Zinc(II) hydrolysis constants (log β) at different [NaCl] (M) at T = 298.1 K.

Equilibrium	0.05	0.15	0.30	0.70	1.00
$Zn^{2+} + 2OH^- = [Zn(OH)_2]$	15.40±0.06	15.76±0.01	15.77±0.01	15.93±0.01	16.05±0.01
$Zn^{2+} + 3OH^- = [Zn(OH)_3]^-$	25.16±0.09	26.15±0.04	26.10±0.03	26.51±0.04	26.56±0.03

Table S4. Intrinsic stability constants (log β^0) for zinc(II)-PYR species calculated using an indirect method on data at different ionic strength (M NaCl) and a direct method on the full ionic strength dataset.

	**Direct**	**Indirect**				
		**0.05**	**0.15**	**0.30**	**0.70**	**1.00**
HL^-	12.49 ±0.009	12.50±0.03	12.45±0.01	12.48±0.01	12.46±0.01	12.20±0.01
H_2L	21.87±0.001	21.61±0.07	21.48±0.02	21.45±0.02	21.4±0.02	21.18±0.02
ZnL	9.90±0.001	9.97±0.03	9.85±0.02	9.65±0.01	9.50±0.01	9.05±01
ZnL_2^{2-}	17.14±0.007	16.54±0.04	15.70±0.02	15.03±0.01	14.84±0.01	14.42±0.01

Table S5. Concentration of zinc(II)-PYR complexes and calculated binding efficiencies (BE) at different [NaCl] (M) between pH 5 – 9.

pH	0.005	0.010	$BE_{0.010}$	0.020	$BE_{0.020}$	0.040	$BE_{0.040}$	0.080	$BE_{0.080}$	0.160	$BE_{0.160}$	0.320	$BE_{0.320}$	0.640	$BE_{0.640}$	1.280	$BE_{1.280}$
5	6.3E-08	6.0E-08	9.5E-01	5.6E-08	8.9E-01	5.0E-08	7.9E-01	4.4E-08	6.9E-01	3.7E-08	5.9E-01	3.0E-08	4.7E-01	2.0E-08	3.2E-01	1.1E-08	1.8E-01
5.08	9.1E-08	8.7E-08	9.5E-01	8.1E-08	8.9E-01	7.2E-08	7.9E-01	6.3E-08	6.9E-01	5.4E-08	5.9E-01	4.3E-08	4.7E-01	3.0E-08	3.2E-01	1.7E-08	1.8E-01
5.16	1.3E-07	1.3E-07	9.6E-01	1.2E-07	8.9E-01	1.0E-07	7.9E-01	9.1E-08	6.9E-01	7.8E-08	5.9E-01	6.2E-08	4.7E-01	4.3E-08	3.2E-01	2.4E-08	1.8E-01
5.24	1.9E-07	1.8E-07	9.6E-01	1.7E-07	8.9E-01	1.5E-07	7.9E-01	1.3E-07	6.9E-01	1.1E-07	5.9E-01	8.9E-08	4.7E-01	6.2E-08	3.2E-01	3.5E-08	1.8E-01
5.32	2.8E-07	2.6E-07	9.5E-01	2.5E-07	8.9E-01	2.2E-07	7.9E-01	1.9E-07	6.9E-01	1.6E-07	5.9E-01	1.3E-07	4.7E-01	8.9E-08	3.2E-01	5.0E-08	1.8E-01
5.4	4.0E-07	3.8E-07	9.5E-01	3.5E-07	8.9E-01	3.2E-07	7.9E-01	2.8E-07	6.9E-01	2.3E-07	5.9E-01	1.9E-07	4.7E-01	1.3E-07	3.2E-01	7.2E-08	1.8E-01
5.48	5.8E-07	5.5E-07	9.5E-01	5.1E-07	8.9E-01	4.6E-07	7.9E-01	4.0E-07	6.9E-01	3.4E-07	5.9E-01	2.7E-07	4.7E-01	1.9E-07	3.2E-01	1.0E-07	1.8E-01
5.56	8.3E-07	7.9E-07	9.6E-01	7.4E-07	8.9E-01	6.6E-07	7.9E-01	5.8E-07	6.9E-01	4.9E-07	5.9E-01	3.9E-07	4.7E-01	2.7E-07	3.2E-01	1.5E-07	1.8E-01
5.64	1.2E-06	1.1E-06	9.6E-01	1.1E-06	8.9E-01	9.5E-07	7.9E-01	8.3E-07	6.9E-01	7.1E-07	5.9E-01	5.6E-07	4.7E-01	3.9E-07	3.2E-01	2.2E-07	1.8E-01
5.72	1.7E-06	1.7E-06	9.6E-01	1.5E-06	8.9E-01	1.4E-06	7.9E-01	1.2E-06	6.9E-01	1.0E-06	5.9E-01	8.1E-07	4.7E-01	5.6E-07	3.2E-01	3.2E-07	1.8E-01
5.8	2.5E-06	2.4E-06	9.5E-01	2.2E-06	8.9E-01	2.0E-06	7.9E-01	1.7E-06	6.9E-01	1.5E-06	5.9E-01	1.2E-06	4.7E-01	8.1E-07	3.2E-01	4.6E-07	1.8E-01
5.88	3.6E-06	3.5E-06	9.5E-01	3.2E-06	8.9E-01	2.9E-06	7.9E-01	2.5E-06	6.9E-01	2.1E-06	5.9E-01	1.7E-06	4.7E-01	1.2E-06	3.2E-01	6.6E-07	1.8E-01
5.96	5.2E-06	5.0E-06	9.6E-01	4.7E-06	8.9E-01	4.2E-06	7.9E-01	3.6E-06	6.9E-01	3.1E-06	5.9E-01	2.5E-06	4.7E-01	1.7E-06	3.2E-01	9.5E-07	1.8E-01
6.04	7.6E-06	7.2E-06	9.6E-01	6.8E-06	8.9E-01	6.0E-06	7.9E-01	5.2E-06	6.9E-01	4.5E-06	5.9E-01	3.5E-06	4.7E-01	2.5E-06	3.2E-01	1.4E-06	1.8E-01
6.12	1.1E-05	1.0E-05	9.6E-01	9.8E-06	8.9E-01	8.7E-06	7.9E-01	7.6E-06	6.9E-01	6.5E-06	5.9E-01	5.1E-06	4.7E-01	3.5E-06	3.2E-01	2.0E-06	1.8E-01
6.2	1.6E-05	1.5E-05	9.6E-01	1.4E-05	8.9E-01	1.3E-05	7.9E-01	1.1E-05	6.9E-01	9.3E-06	5.9E-01	7.4E-06	4.7E-01	5.1E-06	3.2E-01	2.9E-06	1.8E-01
6.28	2.3E-05	2.2E-05	9.6E-01	2.0E-05	8.9E-01	1.8E-05	7.9E-01	1.6E-05	6.9E-01	1.3E-05	5.9E-01	1.1E-05	4.7E-01	7.4E-06	3.2E-01	4.2E-06	1.8E-01
6.36	3.3E-05	3.2E-05	9.5E-01	2.9E-05	8.9E-01	2.6E-05	7.9E-01	2.3E-05	6.9E-01	1.9E-05	5.9E-01	1.5E-05	4.7E-01	1.1E-05	3.2E-01	6.0E-06	1.8E-01
6.44	4.8E-05	4.6E-05	9.5E-01	4.3E-05	8.9E-01	3.8E-05	7.9E-01	3.3E-05	6.9E-01	2.8E-05	5.9E-01	2.2E-05	4.7E-01	1.5E-05	3.2E-01	8.7E-06	1.8E-01
6.52	6.9E-05	6.6E-05	9.5E-01	6.2E-05	8.9E-01	5.5E-05	7.9E-01	4.8E-05	6.9E-01	4.1E-05	5.9E-01	3.2E-05	4.7E-01	2.2E-05	3.2E-01	1.3E-05	1.8E-01
6.6	1.0E-04	9.5E-05	9.5E-01	8.9E-05	8.9E-01	7.9E-05	7.9E-01	6.9E-05	6.9E-01	5.9E-05	5.9E-01	4.7E-05	4.7E-01	3.2E-05	3.2E-01	1.8E-05	1.8E-01
6.68	1.4E-04	1.4E-04	9.5E-01	1.3E-04	8.9E-01	1.1E-04	7.9E-01	1.0E-04	6.9E-01	8.5E-05	5.9E-01	6.7E-05	4.7E-01	4.7E-05	3.2E-01	2.6E-05	1.8E-01
6.76	2.1E-04	2.0E-04	9.5E-01	1.9E-04	8.9E-01	1.7E-04	7.9E-01	1.4E-04	6.9E-01	1.2E-04	5.9E-01	9.7E-05	4.7E-01	6.7E-05	3.2E-01	3.8E-05	1.8E-01
6.84	3.0E-04	2.9E-04	9.5E-01	2.7E-04	8.9E-01	2.4E-04	7.9E-01	2.1E-04	6.9E-01	1.8E-04	5.9E-01	1.4E-04	4.7E-01	9.7E-05	3.2E-01	5.5E-05	1.8E-01

6.92	4.3E-04	4.2E-04	9.5E-01	3.9E-04	8.9E-01	3.5E-04	7.9E-01	3.0E-04	6.9E-01	2.6E-04	5.9E-01	2.0E-04	4.7E-01	1.4E-04	3.2E-01	7.9E-05	1.8E-01
7	6.3E-04	6.0E-04	9.5E-01	5.6E-04	8.9E-01	5.0E-04	7.9E-01	4.3E-04	6.9E-01	3.7E-04	5.9E-01	2.9E-04	4.7E-01	2.0E-04	3.2E-01	1.1E-04	1.8E-01
7.08	9.1E-04	8.7E-04	9.5E-01	8.1E-04	8.9E-01	7.2E-04	7.9E-01	6.3E-04	6.9E-01	5.3E-04	5.9E-01	4.2E-04	4.7E-01	2.9E-04	3.2E-01	1.6E-04	1.8E-01
7.16	1.3E-03	1.3E-03	9.5E-01	1.2E-03	8.9E-01	1.0E-03	7.9E-01	9.0E-04	6.9E-01	7.7E-04	5.9E-01	6.1E-04	4.7E-01	4.2E-04	3.2E-01	2.4E-04	1.8E-01
7.24	1.9E-03	1.8E-03	9.5E-01	1.7E-03	8.9E-01	1.5E-03	7.9E-01	1.3E-03	6.9E-01	1.1E-03	5.9E-01	8.8E-04	4.7E-01	6.1E-04	3.2E-01	3.4E-04	1.8E-01
7.32	2.7E-03	2.6E-03	9.5E-01	2.4E-03	8.9E-01	2.2E-03	7.9E-01	1.9E-03	6.9E-01	1.6E-03	5.9E-01	1.3E-03	4.7E-01	8.8E-04	3.2E-01	4.9E-04	1.8E-01
7.4	3.9E-03	3.7E-03	9.5E-01	3.5E-03	8.9E-01	3.1E-03	7.9E-01	2.7E-03	6.9E-01	2.3E-03	5.9E-01	1.8E-03	4.7E-01	1.3E-03	3.2E-01	7.1E-04	1.8E-01
7.48	5.7E-03	5.4E-03	9.5E-01	5.0E-03	8.9E-01	4.5E-03	7.9E-01	3.9E-03	6.9E-01	3.3E-03	5.9E-01	2.6E-03	4.7E-01	1.8E-03	3.2E-01	1.0E-03	1.8E-01
7.56	8.1E-03	7.8E-03	9.5E-01	7.2E-03	8.9E-01	6.4E-03	7.9E-01	5.6E-03	6.9E-01	4.8E-03	5.9E-01	3.8E-03	4.7E-01	2.6E-03	3.2E-01	1.5E-03	1.8E-01
7.64	1.2E-02	1.1E-02	9.5E-01	1.0E-02	8.9E-01	9.3E-03	7.9E-01	8.1E-03	6.9E-01	6.8E-03	5.9E-01	5.4E-03	4.7E-01	3.8E-03	3.2E-01	2.1E-03	1.8E-01
7.72	1.7E-02	1.6E-02	9.6E-01	1.5E-02	8.9E-01	1.3E-02	7.9E-01	1.2E-02	6.9E-01	9.8E-03	5.9E-01	7.8E-03	4.7E-01	5.4E-03	3.2E-01	3.0E-03	1.8E-01
7.8	2.4E-02	2.3E-02	9.6E-01	2.1E-02	8.9E-01	1.9E-02	7.9E-01	1.7E-02	6.9E-01	1.4E-02	5.9E-01	1.1E-02	4.7E-01	7.7E-03	3.2E-01	4.4E-03	1.8E-01
7.88	3.4E-02	3.2E-02	9.6E-01	3.0E-02	8.9E-01	2.7E-02	8.0E-01	2.4E-02	6.9E-01	2.0E-02	5.9E-01	1.6E-02	4.7E-01	1.1E-02	3.3E-01	6.2E-03	1.8E-01
7.96	4.8E-02	4.6E-02	9.6E-01	4.3E-02	8.9E-01	3.8E-02	8.0E-01	3.3E-02	7.0E-01	2.8E-02	5.9E-01	2.3E-02	4.7E-01	1.6E-02	3.3E-01	8.9E-03	1.9E-01
8.04	6.7E-02	6.4E-02	9.6E-01	6.0E-02	8.9E-01	5.4E-02	8.0E-01	4.7E-02	7.0E-01	4.0E-02	6.0E-01	3.2E-02	4.7E-01	2.2E-02	3.3E-01	1.3E-02	1.9E-01
8.12	9.4E-02	9.0E-02	9.6E-01	8.4E-02	9.0E-01	7.5E-02	8.0E-01	6.6E-02	7.0E-01	5.6E-02	6.0E-01	4.5E-02	4.8E-01	3.1E-02	3.4E-01	1.8E-02	1.9E-01
8.2	1.3E-01	1.2E-01	9.6E-01	1.2E-01	9.0E-01	1.0E-01	8.1E-01	9.1E-02	7.1E-01	7.8E-02	6.1E-01	6.3E-02	4.9E-01	4.4E-02	3.4E-01	2.5E-02	2.0E-01
8.28	1.7E-01	1.7E-01	9.6E-01	1.6E-01	9.0E-01	1.4E-01	8.2E-01	1.2E-01	7.2E-01	1.1E-01	6.2E-01	8.7E-02	5.0E-01	6.1E-02	3.5E-01	3.6E-02	2.1E-01
8.36	2.3E-01	2.2E-01	9.6E-01	2.1E-01	9.1E-01	1.9E-01	8.3E-01	1.7E-01	7.3E-01	1.4E-01	6.3E-01	1.2E-01	5.2E-01	8.4E-02	3.7E-01	5.0E-02	2.2E-01
8.44	3.0E-01	2.9E-01	9.7E-01	2.7E-01	9.2E-01	2.5E-01	8.4E-01	2.2E-01	7.5E-01	1.9E-01	6.5E-01	1.6E-01	5.4E-01	1.1E-01	3.9E-01	6.8E-02	2.3E-01
8.52	3.7E-01	3.6E-01	9.7E-01	3.4E-01	9.2E-01	3.2E-01	8.5E-01	2.8E-01	7.7E-01	2.5E-01	6.7E-01	2.1E-01	5.6E-01	1.5E-01	4.1E-01	9.3E-02	2.5E-01
8.6	4.5E-01	4.4E-01	9.7E-01	4.2E-01	9.3E-01	3.9E-01	8.7E-01	3.6E-01	7.9E-01	3.2E-01	7.0E-01	2.7E-01	5.9E-01	2.0E-01	4.4E-01	1.2E-01	2.8E-01
8.68	5.4E-01	5.2E-01	9.8E-01	5.0E-01	9.4E-01	4.7E-01	8.8E-01	4.3E-01	8.1E-01	3.9E-01	7.3E-01	3.3E-01	6.3E-01	2.6E-01	4.8E-01	1.6E-01	3.1E-01
8.76	6.2E-01	6.0E-01	9.8E-01	5.8E-01	9.5E-01	5.5E-01	9.0E-01	5.1E-01	8.4E-01	4.7E-01	7.6E-01	4.1E-01	6.6E-01	3.2E-01	5.2E-01	2.1E-01	3.5E-01
8.84	6.9E-01	6.8E-01	9.8E-01	6.6E-01	9.6E-01	6.3E-01	9.1E-01	5.9E-01	8.6E-01	5.5E-01	7.9E-01	4.9E-01	7.0E-01	3.9E-01	5.7E-01	2.7E-01	3.9E-01
8.92	7.5E-01	7.4E-01	9.9E-01	7.3E-01	9.6E-01	7.0E-01	9.3E-01	6.7E-01	8.8E-01	6.2E-01	8.2E-01	5.6E-01	7.4E-01	4.7E-01	6.2E-01	3.3E-01	4.4E-01

9	8.1E-01	8.0E-01	9.9E-01	7.9E-01	9.7E-01	7.6E-01	9.4E-01	7.3E-01	9.0E-01	6.9E-01	8.5E-01	6.3E-01	7.8E-01	5.4E-01	6.7E-01	4.0E-01	4.9E-01

Table S6. Concentration of zinc(II)-GLY complexes and calculated binding efficiencies (BE) at different [NaCl] (M) between pH 5 – 9.

pH	0.005	0.010	$BE_{0.010}$	0.020	$BE_{0.020}$	0.040	$BE_{0.040}$	0.080	$BE_{0.080}$	0.160	$BE_{0.160}$	0.320	$BE_{0.320}$	0.640	$BE_{0.640}$	1.280	$BE_{1.280}$
5	6.8E-03	6.0E-03	8.8E-01	5.1E-03	7.5E-01	4.1E-03	6.0E-01	2.9E-03	4.3E-01	1.8E-03	2.6E-01	8.2E-04	1.2E-01	2.2E-04	3.3E-02	2.1E-05	3.1E-03
5.08	6.9E-03	6.1E-03	8.8E-01	5.2E-03	7.5E-01	4.2E-03	6.0E-01	3.0E-03	4.3E-01	1.8E-03	2.6E-01	8.3E-04	1.2E-01	2.2E-04	3.2E-02	2.1E-05	3.1E-03
5.16	7.0E-03	6.2E-03	8.8E-01	5.3E-03	7.5E-01	4.2E-03	6.0E-01	3.0E-03	4.3E-01	1.8E-03	2.6E-01	8.4E-04	1.2E-01	2.3E-04	3.2E-02	2.2E-05	3.1E-03
5.24	7.1E-03	6.2E-03	8.8E-01	5.3E-03	7.5E-01	4.2E-03	6.0E-01	3.0E-03	4.3E-01	1.8E-03	2.6E-01	8.4E-04	1.2E-01	2.3E-04	3.2E-02	2.2E-05	3.1E-03
5.32	7.2E-03	6.3E-03	8.7E-01	5.4E-03	7.5E-01	4.3E-03	6.0E-01	3.0E-03	4.2E-01	1.8E-03	2.6E-01	8.5E-04	1.2E-01	2.3E-04	3.2E-02	2.2E-05	3.0E-03
5.4	7.2E-03	6.3E-03	8.7E-01	5.4E-03	7.5E-01	4.3E-03	6.0E-01	3.1E-03	4.2E-01	1.9E-03	2.6E-01	8.5E-04	1.2E-01	2.3E-04	3.2E-02	2.2E-05	3.0E-03
5.48	7.3E-03	6.4E-03	8.7E-01	5.4E-03	7.5E-01	4.3E-03	5.9E-01	3.1E-03	4.2E-01	1.9E-03	2.6E-01	8.5E-04	1.2E-01	2.3E-04	3.2E-02	2.2E-05	3.0E-03
5.56	7.3E-03	6.4E-03	8.7E-01	5.5E-03	7.5E-01	4.3E-03	5.9E-01	3.1E-03	4.2E-01	1.9E-03	2.6E-01	8.6E-04	1.2E-01	2.3E-04	3.2E-02	2.2E-05	3.0E-03
5.64	7.4E-03	6.4E-03	8.7E-01	5.5E-03	7.5E-01	4.4E-03	5.9E-01	3.1E-03	4.2E-01	1.9E-03	2.5E-01	8.6E-04	1.2E-01	2.3E-04	3.1E-02	2.2E-05	3.0E-03
5.72	7.4E-03	6.4E-03	8.7E-01	5.5E-03	7.4E-01	4.4E-03	5.9E-01	3.1E-03	4.2E-01	1.9E-03	2.5E-01	8.6E-04	1.2E-01	2.3E-04	3.1E-02	2.2E-05	3.0E-03
5.8	7.4E-03	6.5E-03	8.7E-01	5.5E-03	7.4E-01	4.4E-03	5.9E-01	3.1E-03	4.2E-01	1.9E-03	2.5E-01	8.6E-04	1.2E-01	2.3E-04	3.1E-02	2.2E-05	3.0E-03
5.88	7.4E-03	6.5E-03	8.7E-01	5.5E-03	7.4E-01	4.4E-03	5.9E-01	3.1E-03	4.2E-01	1.9E-03	2.5E-01	8.6E-04	1.2E-01	2.3E-04	3.1E-02	2.2E-05	3.0E-03
5.96	7.4E-03	6.5E-03	8.7E-01	5.5E-03	7.4E-01	4.4E-03	5.9E-01	3.1E-03	4.2E-01	1.9E-03	2.5E-01	8.6E-04	1.2E-01	2.3E-04	3.1E-02	2.2E-05	3.0E-03
6.04	7.5E-03	6.5E-03	8.7E-01	5.5E-03	7.4E-01	4.4E-03	5.9E-01	3.1E-03	4.2E-01	1.9E-03	2.5E-01	8.7E-04	1.2E-01	2.3E-04	3.1E-02	2.2E-05	3.0E-03
6.12	7.5E-03	6.5E-03	8.7E-01	5.6E-03	7.4E-01	4.4E-03	5.9E-01	3.1E-03	4.2E-01	1.9E-03	2.5E-01	8.7E-04	1.2E-01	2.3E-04	3.1E-02	2.2E-05	3.0E-03
6.2	7.5E-03	6.5E-03	8.7E-01	5.6E-03	7.4E-01	4.4E-03	5.9E-01	3.1E-03	4.2E-01	1.9E-03	2.5E-01	8.7E-04	1.2E-01	2.3E-04	3.1E-02	2.2E-05	3.0E-03
6.28	7.5E-03	6.5E-03	8.7E-01	5.6E-03	7.4E-01	4.4E-03	5.9E-01	3.1E-03	4.2E-01	1.9E-03	2.5E-01	8.7E-04	1.2E-01	2.3E-04	3.1E-02	2.2E-05	3.0E-03
6.36	7.5E-03	6.5E-03	8.7E-01	5.6E-03	7.4E-01	4.4E-03	5.9E-01	3.1E-03	4.2E-01	1.9E-03	2.5E-01	8.7E-04	1.2E-01	2.3E-04	3.1E-02	2.2E-05	3.0E-03
6.44	7.5E-03	6.5E-03	8.7E-01	5.6E-03	7.4E-01	4.4E-03	5.9E-01	3.1E-03	4.2E-01	1.9E-03	2.5E-01	8.7E-04	1.2E-01	2.3E-04	3.1E-02	2.2E-05	3.0E-03
6.52	7.5E-03	6.5E-03	8.7E-01	5.6E-03	7.4E-01	4.4E-03	5.9E-01	3.1E-03	4.2E-01	1.9E-03	2.5E-01	8.7E-04	1.2E-01	2.3E-04	3.1E-02	2.2E-05	3.0E-03
6.6	7.5E-03	6.5E-03	8.7E-01	5.6E-03	7.4E-01	4.4E-03	5.9E-01	3.1E-03	4.2E-01	1.9E-03	2.5E-01	8.7E-04	1.2E-01	2.3E-04	3.1E-02	2.2E-05	3.0E-03
6.68	7.5E-03	6.5E-03	8.7E-01	5.6E-03	7.4E-01	4.4E-03	5.9E-01	3.1E-03	4.2E-01	1.9E-03	2.5E-01	8.7E-04	1.2E-01	2.3E-04	3.1E-02	2.2E-05	3.0E-03
6.76	7.5E-03	6.5E-03	8.7E-01	5.6E-03	7.4E-01	4.4E-03	5.9E-01	3.1E-03	4.2E-01	1.9E-03	2.5E-01	8.7E-04	1.2E-01	2.3E-04	3.1E-02	2.2E-05	3.0E-03
6.84	7.5E-03	6.6E-03	8.7E-01	5.6E-03	7.4E-01	4.4E-03	5.9E-01	3.1E-03	4.2E-01	1.9E-03	2.5E-01	8.7E-04	1.2E-01	2.3E-04	3.1E-02	2.2E-05	3.0E-03

6.92	7.5E-03	6.6E-03	8.7E-01	5.6E-03	7.4E-01	4.4E-03	5.9E-01	3.1E-03	4.2E-01	1.9E-03	2.5E-01	8.7E-04	1.2E-01	2.3E-04	3.1E-02	2.2E-05	3.0E-03
7	7.5E-03	6.6E-03	8.7E-01	5.6E-03	7.4E-01	4.4E-03	5.9E-01	3.1E-03	4.2E-01	1.9E-03	2.5E-01	8.7E-04	1.2E-01	2.3E-04	3.1E-02	2.2E-05	3.0E-03
7.08	7.5E-03	6.6E-03	8.7E-01	5.6E-03	7.4E-01	4.4E-03	5.9E-01	3.1E-03	4.2E-01	1.9E-03	2.5E-01	8.7E-04	1.2E-01	2.3E-04	3.1E-02	2.2E-05	3.0E-03
7.16	7.5E-03	6.6E-03	8.7E-01	5.6E-03	7.4E-01	4.4E-03	5.9E-01	3.2E-03	4.2E-01	1.9E-03	2.5E-01	8.7E-04	1.2E-01	2.3E-04	3.1E-02	2.2E-05	3.0E-03
7.24	7.5E-03	6.6E-03	8.7E-01	5.6E-03	7.4E-01	4.4E-03	5.9E-01	3.2E-03	4.2E-01	1.9E-03	2.5E-01	8.7E-04	1.2E-01	2.3E-04	3.1E-02	2.2E-05	3.0E-03
7.32	7.5E-03	6.6E-03	8.7E-01	5.6E-03	7.4E-01	4.4E-03	5.9E-01	3.2E-03	4.2E-01	1.9E-03	2.5E-01	8.7E-04	1.2E-01	2.3E-04	3.1E-02	2.2E-05	3.0E-03
7.4	7.5E-03	6.6E-03	8.7E-01	5.6E-03	7.4E-01	4.4E-03	5.9E-01	3.2E-03	4.2E-01	1.9E-03	2.5E-01	8.7E-04	1.2E-01	2.3E-04	3.1E-02	2.2E-05	3.0E-03
7.48	7.5E-03	6.6E-03	8.7E-01	5.6E-03	7.4E-01	4.4E-03	5.9E-01	3.2E-03	4.2E-01	1.9E-03	2.5E-01	8.7E-04	1.2E-01	2.3E-04	3.1E-02	2.2E-05	3.0E-03
7.56	7.5E-03	6.6E-03	8.7E-01	5.6E-03	7.4E-01	4.4E-03	5.9E-01	3.2E-03	4.2E-01	1.9E-03	2.5E-01	8.7E-04	1.2E-01	2.3E-04	3.1E-02	2.2E-05	3.0E-03
7.64	7.5E-03	6.6E-03	8.7E-01	5.6E-03	7.4E-01	4.4E-03	5.9E-01	3.2E-03	4.2E-01	1.9E-03	2.5E-01	8.7E-04	1.2E-01	2.3E-04	3.1E-02	2.2E-05	3.0E-03
7.72	7.5E-03	6.6E-03	8.7E-01	5.6E-03	7.4E-01	4.4E-03	5.9E-01	3.2E-03	4.2E-01	1.9E-03	2.5E-01	8.7E-04	1.2E-01	2.3E-04	3.1E-02	2.2E-05	3.0E-03
7.8	7.5E-03	6.6E-03	8.7E-01	5.6E-03	7.4E-01	4.4E-03	5.9E-01	3.2E-03	4.2E-01	1.9E-03	2.5E-01	8.7E-04	1.2E-01	2.3E-04	3.1E-02	2.2E-05	3.0E-03
7.88	7.5E-03	6.6E-03	8.7E-01	5.6E-03	7.4E-01	4.4E-03	5.9E-01	3.2E-03	4.2E-01	1.9E-03	2.5E-01	8.7E-04	1.2E-01	2.3E-04	3.1E-02	2.2E-05	3.0E-03
7.96	7.5E-03	6.6E-03	8.7E-01	5.6E-03	7.4E-01	4.4E-03	5.9E-01	3.2E-03	4.2E-01	1.9E-03	2.5E-01	8.7E-04	1.2E-01	2.3E-04	3.1E-02	2.2E-05	3.0E-03
8.04	7.5E-03	6.6E-03	8.7E-01	5.6E-03	7.4E-01	4.4E-03	5.9E-01	3.2E-03	4.2E-01	1.9E-03	2.5E-01	8.7E-04	1.2E-01	2.3E-04	3.1E-02	2.2E-05	3.0E-03
8.12	7.5E-03	6.6E-03	8.7E-01	5.6E-03	7.4E-01	4.4E-03	5.9E-01	3.2E-03	4.2E-01	1.9E-03	2.5E-01	8.7E-04	1.2E-01	2.3E-04	3.1E-02	2.2E-05	3.0E-03
8.2	7.5E-03	6.6E-03	8.7E-01	5.6E-03	7.4E-01	4.4E-03	5.9E-01	3.2E-03	4.2E-01	1.9E-03	2.5E-01	8.7E-04	1.2E-01	2.3E-04	3.1E-02	2.2E-05	3.0E-03
8.28	7.5E-03	6.6E-03	8.7E-01	5.6E-03	7.4E-01	4.4E-03	5.9E-01	3.2E-03	4.2E-01	1.9E-03	2.5E-01	8.7E-04	1.2E-01	2.3E-04	3.1E-02	2.2E-05	3.0E-03
8.36	7.5E-03	6.6E-03	8.7E-01	5.6E-03	7.4E-01	4.4E-03	5.9E-01	3.2E-03	4.2E-01	1.9E-03	2.5E-01	8.7E-04	1.2E-01	2.3E-04	3.1E-02	2.2E-05	3.0E-03
8.44	7.5E-03	6.6E-03	8.7E-01	5.6E-03	7.4E-01	4.4E-03	5.9E-01	3.2E-03	4.2E-01	1.9E-03	2.5E-01	8.7E-04	1.2E-01	2.3E-04	3.1E-02	2.2E-05	3.0E-03
8.52	7.5E-03	6.6E-03	8.7E-01	5.6E-03	7.4E-01	4.4E-03	5.9E-01	3.2E-03	4.2E-01	1.9E-03	2.5E-01	8.7E-04	1.2E-01	2.3E-04	3.1E-02	2.2E-05	3.0E-03
8.6	7.5E-03	6.6E-03	8.7E-01	5.6E-03	7.4E-01	4.4E-03	5.9E-01	3.2E-03	4.2E-01	1.9E-03	2.5E-01	8.7E-04	1.2E-01	2.3E-04	3.1E-02	2.2E-05	3.0E-03
8.68	7.5E-03	6.6E-03	8.7E-01	5.6E-03	7.4E-01	4.4E-03	5.9E-01	3.2E-03	4.2E-01	1.9E-03	2.5E-01	8.7E-04	1.2E-01	2.3E-04	3.1E-02	2.2E-05	3.0E-03
8.76	7.5E-03	6.6E-03	8.7E-01	5.6E-03	7.4E-01	4.4E-03	5.9E-01	3.2E-03	4.2E-01	1.9E-03	2.5E-01	8.7E-04	1.2E-01	2.3E-04	3.1E-02	2.2E-05	3.0E-03
8.84	7.5E-03	6.6E-03	8.7E-01	5.6E-03	7.4E-01	4.4E-03	5.9E-01	3.2E-03	4.2E-01	1.9E-03	2.5E-01	8.7E-04	1.2E-01	2.3E-04	3.1E-02	2.2E-05	3.0E-03
8.92	7.5E-03	6.6E-03	8.7E-01	5.6E-03	7.4E-01	4.4E-03	5.9E-01	3.2E-03	4.2E-01	1.9E-03	2.5E-01	8.7E-04	1.2E-01	2.3E-04	3.1E-02	2.2E-05	3.0E-03

9	7.5E-03	6.6E-03	8.7E-01	5.6E-03	7.4E-01	4.4E-03	5.9E-01	3.2E-03	4.2E-01	1.9E-03	2.5E-01	8.7E-04	1.2E-01	2.3E-04	3.1E-02	2.2E-05	3.0E-03

Table S7. Concentration of zinc(II)-AHA complexes and calculated binding efficiencies (BE) at different [NaCl] (M) between pH 5 – 9.

pH	0.005	0.010	$BE_{0.010}$	0.020	$BE_{0.020}$	0.040	$BE_{0.040}$	0.080	$BE_{0.080}$	0.160	$BE_{0.160}$	0.320	$BE_{0.320}$	0.640	$BE_{0.640}$	1.280	$BE_{1.280}$
5.00	1.5E-04	1.5E-04	9.6E-01	1.3E-04	8.7E-01	1.3E-04	8.1E-01	1.1E-04	7.1E-01	9.5E-05	6.2E-01	8.3E-05	5.4E-01	6.6E-05	4.3E-01	5.2E-05	3.4E-01
5.08	1.9E-04	1.8E-04	9.5E-01	1.6E-04	8.7E-01	1.5E-04	8.1E-01	1.3E-04	7.1E-01	1.1E-04	6.2E-01	1.0E-04	5.4E-01	7.9E-05	4.3E-01	6.3E-05	3.4E-01
5.16	2.2E-04	2.1E-04	9.5E-01	1.9E-04	8.7E-01	1.8E-04	8.1E-01	1.6E-04	7.1E-01	1.4E-04	6.2E-01	1.2E-04	5.4E-01	9.5E-05	4.3E-01	7.6E-05	3.4E-01
5.24	2.7E-04	2.6E-04	9.5E-01	2.3E-04	8.7E-01	2.2E-04	8.1E-01	1.9E-04	7.1E-01	1.7E-04	6.2E-01	1.4E-04	5.4E-01	1.1E-04	4.3E-01	9.1E-05	3.4E-01
5.32	3.2E-04	3.1E-04	9.6E-01	2.8E-04	8.7E-01	2.6E-04	8.1E-01	2.3E-04	7.1E-01	2.0E-04	6.2E-01	1.7E-04	5.4E-01	1.4E-04	4.3E-01	1.1E-04	3.4E-01
5.40	3.9E-04	3.7E-04	9.6E-01	3.4E-04	8.7E-01	3.2E-04	8.1E-01	2.8E-04	7.1E-01	2.4E-04	6.2E-01	2.1E-04	5.4E-01	1.7E-04	4.3E-01	1.3E-04	3.4E-01
5.48	4.7E-04	4.5E-04	9.6E-01	4.1E-04	8.7E-01	3.8E-04	8.1E-01	3.3E-04	7.1E-01	2.9E-04	6.2E-01	2.5E-04	5.4E-01	2.0E-04	4.3E-01	1.6E-04	3.4E-01
5.56	5.6E-04	5.4E-04	9.6E-01	4.9E-04	8.7E-01	4.6E-04	8.1E-01	4.0E-04	7.1E-01	3.5E-04	6.2E-01	3.0E-04	5.4E-01	2.4E-04	4.3E-01	1.9E-04	3.4E-01
5.64	6.8E-04	6.5E-04	9.6E-01	5.9E-04	8.7E-01	5.5E-04	8.1E-01	4.8E-04	7.1E-01	4.2E-04	6.2E-01	3.6E-04	5.4E-01	2.9E-04	4.3E-01	2.3E-04	3.4E-01
5.72	8.1E-04	7.8E-04	9.6E-01	7.1E-04	8.7E-01	6.6E-04	8.1E-01	5.8E-04	7.1E-01	5.0E-04	6.2E-01	4.4E-04	5.4E-01	3.5E-04	4.3E-01	2.8E-04	3.4E-01
5.80	9.8E-04	9.3E-04	9.6E-01	8.5E-04	8.7E-01	7.9E-04	8.1E-01	6.9E-04	7.1E-01	6.0E-04	6.2E-01	5.2E-04	5.4E-01	4.2E-04	4.3E-01	3.3E-04	3.4E-01
5.88	1.2E-03	1.1E-03	9.5E-01	1.0E-03	8.7E-01	9.5E-04	8.1E-01	8.3E-04	7.1E-01	7.2E-04	6.2E-01	6.3E-04	5.4E-01	5.0E-04	4.3E-01	4.0E-04	3.4E-01
5.96	1.4E-03	1.3E-03	9.5E-01	1.2E-03	8.7E-01	1.1E-03	8.1E-01	1.0E-03	7.1E-01	8.7E-04	6.2E-01	7.6E-04	5.4E-01	6.0E-04	4.3E-01	4.8E-04	3.4E-01
6.04	1.7E-03	1.6E-03	9.6E-01	1.5E-03	8.7E-01	1.4E-03	8.1E-01	1.2E-03	7.1E-01	1.0E-03	6.2E-01	9.1E-04	5.4E-01	7.2E-04	4.3E-01	5.7E-04	3.4E-01
6.12	2.0E-03	1.9E-03	9.5E-01	1.8E-03	8.7E-01	1.7E-03	8.1E-01	1.4E-03	7.1E-01	1.3E-03	6.2E-01	1.1E-03	5.4E-01	8.7E-04	4.3E-01	6.9E-04	3.4E-01
6.20	2.4E-03	2.3E-03	9.6E-01	2.1E-03	8.7E-01	2.0E-03	8.1E-01	1.7E-03	7.1E-01	1.5E-03	6.2E-01	1.3E-03	5.4E-01	1.0E-03	4.3E-01	8.3E-04	3.4E-01
6.28	2.9E-03	2.8E-03	9.6E-01	2.6E-03	8.7E-01	2.4E-03	8.1E-01	2.1E-03	7.1E-01	1.8E-03	6.2E-01	1.6E-03	5.4E-01	1.3E-03	4.3E-01	1.0E-03	3.4E-01
6.36	3.5E-03	3.4E-03	9.6E-01	3.1E-03	8.7E-01	2.9E-03	8.1E-01	2.5E-03	7.1E-01	2.2E-03	6.2E-01	1.9E-03	5.4E-01	1.5E-03	4.3E-01	1.2E-03	3.4E-01
6.44	4.3E-03	4.1E-03	9.6E-01	3.7E-03	8.7E-01	3.5E-03	8.1E-01	3.0E-03	7.1E-01	2.6E-03	6.2E-01	2.3E-03	5.4E-01	1.8E-03	4.3E-01	1.4E-03	3.4E-01
6.52	5.1E-03	4.9E-03	9.6E-01	4.5E-03	8.7E-01	4.2E-03	8.1E-01	3.6E-03	7.1E-01	3.2E-03	6.2E-01	2.8E-03	5.4E-01	2.2E-03	4.3E-01	1.7E-03	3.4E-01
6.60	6.1E-03	5.9E-03	9.6E-01	5.4E-03	8.7E-01	5.0E-03	8.2E-01	4.4E-03	7.1E-01	3.8E-03	6.2E-01	3.3E-03	5.4E-01	2.6E-03	4.3E-01	2.1E-03	3.4E-01
6.68	7.4E-03	7.0E-03	9.6E-01	6.4E-03	8.7E-01	6.0E-03	8.2E-01	5.2E-03	7.1E-01	4.6E-03	6.2E-01	4.0E-03	5.4E-01	3.2E-03	4.3E-01	2.5E-03	3.4E-01
6.76	8.8E-03	8.5E-03	9.6E-01	7.7E-03	8.7E-01	7.2E-03	8.2E-01	6.3E-03	7.1E-01	5.5E-03	6.2E-01	4.8E-03	5.4E-01	3.8E-03	4.3E-01	3.0E-03	3.4E-01
6.84	1.1E-02	1.0E-02	9.6E-01	9.3E-03	8.7E-01	8.7E-03	8.2E-01	7.6E-03	7.1E-01	6.6E-03	6.2E-01	5.8E-03	5.4E-01	4.6E-03	4.3E-01	3.6E-03	3.4E-01

6.92	1.3E-02	1.2E-02	9.6E-01	1.1E-02	8.7E-01	1.0E-02	8.2E-01	9.1E-03	7.1E-01	8.0E-03	6.2E-01	6.9E-03	5.4E-01	5.5E-03	4.3E-01	4.3E-03	3.4E-01
7.00	1.5E-02	1.5E-02	9.6E-01	1.3E-02	8.8E-01	1.3E-02	8.2E-01	1.1E-02	7.2E-01	9.6E-03	6.3E-01	8.3E-03	5.4E-01	6.6E-03	4.3E-01	5.2E-03	3.4E-01
7.08	1.8E-02	1.8E-02	9.6E-01	1.6E-02	8.8E-01	1.5E-02	8.2E-01	1.3E-02	7.2E-01	1.2E-02	6.3E-01	1.0E-02	5.5E-01	7.9E-03	4.3E-01	6.2E-03	3.4E-01
7.16	2.2E-02	2.1E-02	9.6E-01	1.9E-02	8.8E-01	1.8E-02	8.2E-01	1.6E-02	7.2E-01	1.4E-02	6.3E-01	1.2E-02	5.5E-01	9.5E-03	4.3E-01	7.4E-03	3.4E-01
7.24	2.6E-02	2.5E-02	9.6E-01	2.3E-02	8.8E-01	2.2E-02	8.2E-01	1.9E-02	7.2E-01	1.7E-02	6.3E-01	1.4E-02	5.5E-01	1.1E-02	4.3E-01	8.9E-03	3.4E-01
7.32	3.1E-02	3.0E-02	9.6E-01	2.8E-02	8.8E-01	2.6E-02	8.2E-01	2.3E-02	7.2E-01	2.0E-02	6.4E-01	1.7E-02	5.5E-01	1.4E-02	4.3E-01	1.1E-02	3.4E-01
7.40	3.8E-02	3.6E-02	9.6E-01	3.3E-02	8.8E-01	3.1E-02	8.3E-01	2.7E-02	7.3E-01	2.4E-02	6.4E-01	2.1E-02	5.6E-01	1.6E-02	4.4E-01	1.3E-02	3.4E-01
7.48	4.5E-02	4.3E-02	9.6E-01	4.0E-02	8.8E-01	3.7E-02	8.3E-01	3.3E-02	7.3E-01	2.9E-02	6.4E-01	2.5E-02	5.6E-01	2.0E-02	4.4E-01	1.5E-02	3.4E-01
7.56	5.4E-02	5.1E-02	9.6E-01	4.7E-02	8.8E-01	4.5E-02	8.3E-01	4.0E-02	7.4E-01	3.5E-02	6.5E-01	3.0E-02	5.6E-01	2.4E-02	4.4E-01	1.8E-02	3.4E-01
7.64	6.4E-02	6.1E-02	9.6E-01	5.7E-02	8.9E-01	5.3E-02	8.4E-01	4.7E-02	7.4E-01	4.2E-02	6.5E-01	3.6E-02	5.7E-01	2.8E-02	4.4E-01	2.2E-02	3.4E-01
7.72	7.6E-02	7.3E-02	9.6E-01	6.8E-02	8.9E-01	6.4E-02	8.4E-01	5.7E-02	7.5E-01	5.0E-02	6.6E-01	4.4E-02	5.7E-01	3.4E-02	4.5E-01	2.6E-02	3.4E-01
7.80	9.0E-02	8.7E-02	9.6E-01	8.0E-02	8.9E-01	7.6E-02	8.5E-01	6.8E-02	7.6E-01	6.0E-02	6.7E-01	5.2E-02	5.8E-01	4.1E-02	4.5E-01	3.1E-02	3.4E-01
7.88	1.1E-01	1.0E-01	9.6E-01	9.6E-02	9.0E-01	9.1E-02	8.5E-01	8.2E-02	7.7E-01	7.3E-02	6.8E-01	6.3E-02	5.9E-01	4.8E-02	4.5E-01	3.6E-02	3.4E-01
7.96	1.3E-01	1.2E-01	9.7E-01	1.1E-01	9.0E-01	1.1E-01	8.6E-01	9.8E-02	7.7E-01	8.7E-02	6.9E-01	7.5E-02	6.0E-01	5.8E-02	4.6E-01	4.3E-02	3.4E-01
8.04	1.5E-01	1.4E-01	9.7E-01	1.3E-01	9.1E-01	1.3E-01	8.7E-01	1.2E-01	7.9E-01	1.0E-01	7.1E-01	9.0E-02	6.1E-01	6.9E-02	4.7E-01	5.1E-02	3.4E-01
8.12	1.7E-01	1.7E-01	9.7E-01	1.6E-01	9.1E-01	1.5E-01	8.8E-01	1.4E-01	8.0E-01	1.2E-01	7.2E-01	1.1E-01	6.2E-01	8.2E-02	4.7E-01	5.9E-02	3.4E-01
8.20	2.0E-01	2.0E-01	9.7E-01	1.9E-01	9.2E-01	1.8E-01	8.9E-01	1.6E-01	8.1E-01	1.5E-01	7.4E-01	1.3E-01	6.4E-01	9.8E-02	4.8E-01	7.0E-02	3.4E-01
8.28	2.4E-01	2.3E-01	9.8E-01	2.2E-01	9.3E-01	2.1E-01	9.0E-01	2.0E-01	8.3E-01	1.8E-01	7.6E-01	1.5E-01	6.5E-01	1.2E-01	4.9E-01	8.1E-02	3.4E-01
8.36	2.7E-01	2.7E-01	9.8E-01	2.5E-01	9.3E-01	2.5E-01	9.1E-01	2.3E-01	8.5E-01	2.1E-01	7.8E-01	1.8E-01	6.7E-01	1.4E-01	5.0E-01	9.4E-02	3.5E-01
8.44	3.1E-01	3.1E-01	9.8E-01	2.9E-01	9.4E-01	2.9E-01	9.2E-01	2.7E-01	8.7E-01	2.5E-01	8.0E-01	2.2E-01	6.9E-01	1.6E-01	5.2E-01	1.1E-01	3.5E-01
8.52	3.5E-01	3.5E-01	9.8E-01	3.4E-01	9.5E-01	3.3E-01	9.3E-01	3.1E-01	8.8E-01	2.9E-01	8.2E-01	2.5E-01	7.2E-01	1.9E-01	5.3E-01	1.2E-01	3.5E-01
8.60	4.0E-01	4.0E-01	9.9E-01	3.8E-01	9.6E-01	3.8E-01	9.4E-01	3.6E-01	9.0E-01	3.4E-01	8.4E-01	3.0E-01	7.4E-01	2.2E-01	5.5E-01	1.4E-01	3.5E-01
8.68	4.5E-01	4.4E-01	9.9E-01	4.3E-01	9.6E-01	4.3E-01	9.6E-01	4.1E-01	9.2E-01	3.9E-01	8.7E-01	3.4E-01	7.6E-01	2.6E-01	5.7E-01	1.6E-01	3.6E-01
8.76	5.0E-01	5.0E-01	9.9E-01	4.9E-01	9.7E-01	4.8E-01	9.7E-01	4.7E-01	9.4E-01	4.5E-01	8.9E-01	4.0E-01	7.9E-01	3.0E-01	5.9E-01	1.8E-01	3.6E-01
8.84	5.5E-01	5.5E-01	9.9E-01	5.4E-01	9.8E-01	5.4E-01	9.8E-01	5.3E-01	9.6E-01	5.0E-01	9.1E-01	4.5E-01	8.1E-01	3.4E-01	6.2E-01	2.0E-01	3.7E-01
8.92	6.0E-01	6.0E-01	1.0E+00	5.9E-01	9.9E-01	5.9E-01	9.9E-01	5.8E-01	9.7E-01	5.6E-01	9.3E-01	5.1E-01	8.4E-01	3.9E-01	6.4E-01	2.3E-01	3.8E-01

9.00	6.5E-01	6.5E-01	1.0E+00	6.5E-01	9.9E-01	6.5E-01	9.9E-01	6.4E-01	9.8E-01	6.2E-01	9.5E-01	5.6E-01	8.6E-01	4.4E-01	6.7E-01	2.6E-01	3.9E-01

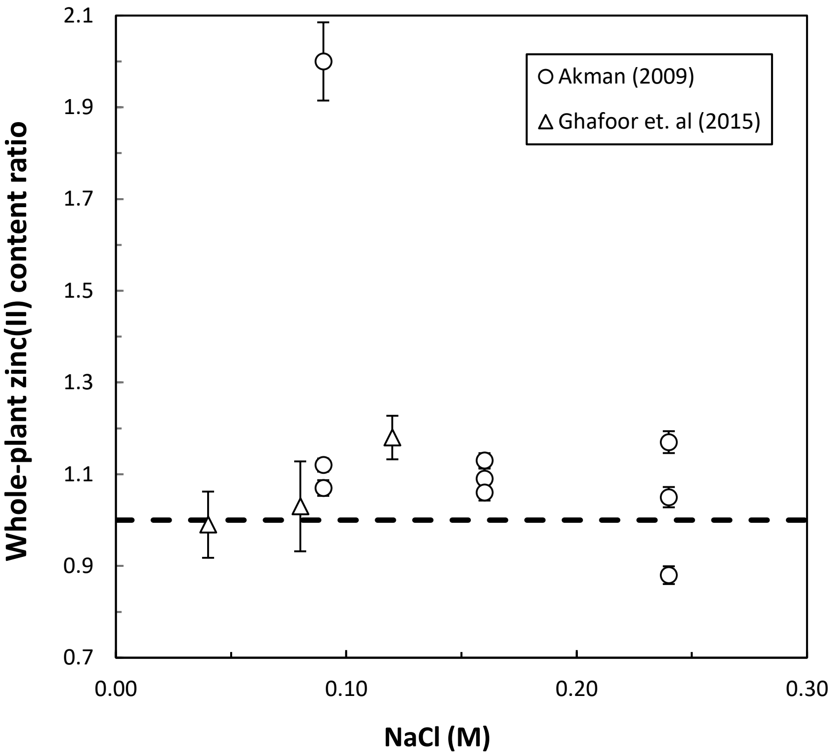

Figure S1. Whole-plant zinc(II) content ratio for barley plants grown under NaCl stress in soil or hydroponic solutions without initial micronutrient deficiency (*i.e.,* no micronutrient limitation prior to salinization). This ratio is calculated by dividing the whole-plant zinc(II) content of the plant grown under salt stress by the whole-plant zinc(II) content of its respective control grown without NaCl added to the growth medium. The dashed line indicates where the ratio is equal to unity. For details of the cultivar studied and collated literature data for specific plant organs refer to Table S1.

Note S1. Matlab code for zinc(II) binding efficiency contour plots.

```
%Read in salinity, pH and binding efficiency data
data = load('FunctionalGroup_A.txt');


x=data(:,1);
y=data(:,2);
z=data(:,3);


%Create interpolant
F = TriScatteredInterp(x,y,z);


[qx qy] = meshgrid(min(x):0.01:max(x),min(y):0.01:max(y));


qz = F(qx,qy);



%Plot and label figure
contourf(qx,qy,qz,'--');


xlabel('pH');


ylabel('NaCl (M)');


xlim([5 9]);


ylim([0.005 1]);


caxis([0, 1]);
```

References

1. Akman, Z. Effects of Plant Growth Regulators on Nutrient Content of Young Wheat and Barley Plants under Saline Conditions. *J. Ani. Vet. Sci.* **2009**, 8(10), 2018-2021.

2. Ali, S.; Cai, S.; Zeng, F.; Qiu, B.; Zhang, G. Effect of salinity and hexavalent chromium stresses on uptake and accumulation of mineral elements in barley genotypes differing in salt tolerance. *J. Plant Nut.* **2012** 35(6), 827-839.

3. Ghafoor, K.; AL-Juhaimi, F.; Ozcan, M. M.; Jahurul, M. H. A. Some Nutritional Characteristics and Mineral Contents in Barley (Hordeum Vulgare L.) Seeds Cultivated under Salt Stress. *Qual. Assur. Saf. Crop. Foods* **2015**, *7* (3), 363–368.

4. Pérez-López, U.; Miranda-Apodaca, J.; Mena-Petite, A.; Muñoz-Rueda, A. Responses of nutrient dynamics in barley seedlings to the interaction of salinity and carbon dioxide enrichment. *J. Env. Exp. Bot.* **2014,** 99, 86-99.

5. Northover, G.H.R.; Mao, Y.; Hanif MD.; Blasco, S.; Vilar, R.; Garcia-Espana, E.; Weiss, D.J. The control of pH and ionic strength gradients on the interaction of low-molecular-weight organic acids and siderophores. *ChemRxiv.* **2021.** *Preprint.*
